# Enhancing RNA base editing on mammalian transcripts with small nuclear RNAs

**DOI:** 10.1038/s41589-025-02026-8

**Published:** 2025-09-18

**Authors:** Aaron A. Smargon, Deepak Pant, Trent A. Gomberg, Christian Fagre, Sofia Glynne, Johnathan Nguyen, Jack T. Naritomi, Wendy V. Gilbert, Gene W. Yeo

**Affiliations:** 1 Department of Cellular and Molecular Medicine, University of California, San Diego, La Jolla, CA USA; 2 Sanford Stem Cell Institute Innovation Center and Sanford Consortium for Regenerative Medicine, La Jolla, CA USA; 3 Institute for Genomic Medicine, University of California, San Diego, La Jolla, CA USA; 4 Biological Sciences Graduate Program, University of California, San Diego, La Jolla, CA USA; 5 Biomedical Sciences Graduate Program, University of California, San Diego, La Jolla, CA USA; 6 Department of Molecular Biophysics and Biochemistry, Yale University, New Haven, CT USA; 7 Center for RNA Technologies and Therapeutics, University of California, San Diego, La Jolla, CA USA

**Keywords:** RNA, Chemical modification, Non-coding RNAs

## Abstract

Endogenous uridine-rich small nuclear RNAs (U snRNAs) form RNA–protein complexes to process eukaryotic pre-mRNA into mRNA. Previous studies have demonstrated programmable U snRNA guide-targeted exon inclusion and exclusion. Here we investigated whether snRNAs can also enhance RNA base editing over state-of-the-art RNA-targeting technologies in human cells. Compared with adenosine deaminase acting on RNA (ADAR)-recruiting circular RNAs, we find that guided A>I snRNAs consistently increase adenosine-to-inosine editing for higher exon count genes, perturb substantially fewer off-target genes and localize more persistently to the nucleus where ADAR is expressed. A>I snRNAs also more efficiently edit long noncoding RNAs and pre-mRNA 3′ splice sites to promote splicing changes. Lastly, snRNA–H/ACA box snoRNA fusions (U>Ψ snRNAs) increase targeted RNA pseudouridylation without DKC1 overexpression, facilitating improved *CFTR* rescue from nonsense-mediated mRNA decay in a cystic fibrosis human bronchial epithelial cell model. Our results advance the endogenous protein-mediated RNA base editing toolbox and RNA-targeting technologies to treat genetic diseases.

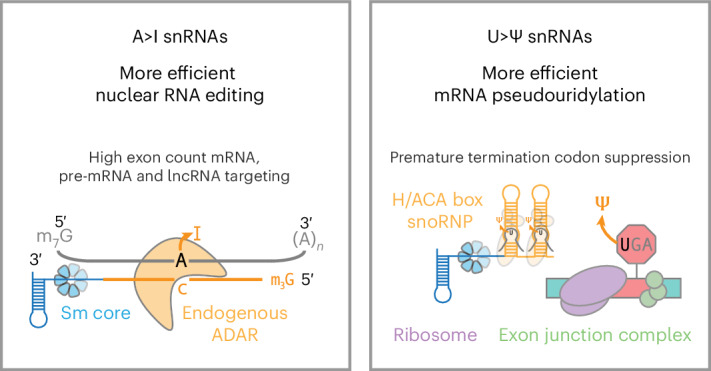

## Abstract (web summary)

Smargon et al. show that small nuclear RNAs can improve the cellular safety and efficacy of endogenous protein-mediated RNA base editing, enhancing nuclear RNA editing and the rescue of premature termination codon disease.

## Main

Recently the gene-editing field has turned from CRISPR (clustered regularly interspaced short palindromic repeats) and other exogenous protein-encoded multicomponent systems toward minimally invasive single-component guided RNA scaffolds that recruit highly expressed endogenous protein machinery to edit genes at the RNA level^1^. Researchers have particularly focused on suppressing in-frame premature termination codons (PTCs) caused by single-base-pair substitution nonsense mutations in coding regions of mRNA transcripts. PTCs, which account for an estimated 10–15% of human genetic diseases such as cystic fibrosis and Hurler syndrome, lead to truncated proteins and subsequent degradation of PTC-harboring mRNAs by nonsense-mediated mRNA decay (NMD)^2^.

Given a clearly defined mechanism, several minimally invasive strategies already exist to treat PTC-associated diseases. More clinically established drugs such as splice-switching antisense oligonucleotides and small molecules could be administered to patients^3–5^, but not all PTC diseases are amenable to exon skipping and PTC suppressor small molecules lack target-site specificity. Similarly, engineered suppressor tRNAs designed to read through PTCs at the translational level could do so at any targeted stop codon context sequence^6,7^. In contrast to these approaches, programmable guided RNA scaffolds that recruit highly expressed endogenous proteins to edit PTC bases directly strike a safer balance between minimal invasiveness and specificity.

One such class of systems recruits endogenous adenosine deaminase acting on RNA (ADAR) enzymes to edit PTC adenosines to inosines. In mammalian cells, active ADAR family members ADAR1 and ADAR2 recognize regions of nuclear double-stranded RNA, primarily at Alu repetitive regions but also in coding regions and even at splice sites^8^. After A>I editing, splicing and translation machinery generally recognizes inosine as its structurally comparable base, guanosine. Leveraging this finding, several groups have encoded a cytosine-mismatch (C-mismatch) guided RNA scaffold of both linear and circular form, which, when hybridized to the RNA sequence surrounding a targeted adenosine, recruits endogenous ADARs that efficiently edit the targeted adenosine opposite the cytosine to inosine^9–11^. While robust editing is possible, ADARs display a strong preference for the UAG motif, with diminished activity for the other PTC sequence contexts of UGA and UAA, in addition to varying by cell type expression^12^.

Another class of systems uses H/ACA box small nucleolar ribonucleoproteins (snoRNPs), which are highly conserved across eukaryotes and catalyze uridine-to-pseudouridine (U>Ψ) editing on small nuclear RNAs (snRNAs), ribosomal RNAs (rRNAs) and some mRNAs^13,14^. In mammalian cells, H/ACA box snoRNAs recruit four core proteins, DKC1, NOP10, NHP2 and GAR1. Together, these proteins edit U>Ψ at a site between two guide-templated regions specified by the H/ACA box snoRNA. Building upon initial work performed in yeast^15^, two groups reprogrammed human H/ACA box snoRNAs to edit U>Ψ at all three PTC sequence contexts (UAG, UGA and UAA), leading to successful translational readthrough^16,17^. While a promising approach, snoRNAs localize predominantly to the nucleolus and not to the nucleoplasm where pre-mRNAs are transcribed and processed into mRNAs, thus limiting their base editing potential.

Although the gene-editing field has evolved beyond CRISPR, important lessons endure from CRISPR’s success. For example, a peptide nuclear localization signal was critical for translation of Cas9 activity from prokaryotic to eukaryotic cells^18,19^. Analogously, we hypothesized that achieving optimal subcellular localization of programmable guided RNA scaffolds could enhance the ability of endogenous protein machinery to perform base editing on target coding RNA transcripts. To test this hypothesis, we selected as a putative RNA nucleoplasm localization signal components of endogenous uridine-rich snRNAs (U snRNAs), which natively recruit protein complexes to process pre-mRNA into mature mRNA and were previously engineered to modulate RNA splicing^20,21^. In this new application, we evaluated the capacity of U snRNAs to enhance endogenous protein-mediated base editing, both A>I and U>Ψ, on mammalian transcripts.

## Results

Preclinical studies using engineered U1 and U7smOPT snRNAs have already shown promise for the inclusion and exclusion of exons in disease rescue^20,21^. In fact, an AAV9-mediated U7smOPT snRNA gene therapy to treat boys with *DMD* exon 2 duplications is currently in phase 1/2 clinical trials (NCT04240314). On the basis of this established track record and the fact that most other U snRNAs are recruited downstream of U1, we concentrated on these two U snRNAs. U1 snRNAs (bound by highly expressed U1A, U1-70K, U1C and members of the Sm core) initiate the major spliceosome to splice introns out of pre-mRNA. Meanwhile, U7 snRNAs initiate the 3′ end processing of nonpolyadenylated histone pre-mRNAs. Researchers previously mutated U7 snRNAs into U7smOPT snRNAs that bind only the Sm core, a key component of the majority of splicing U snRNAs, and not LSm proteins. With backbone sizes of 153 and 45 nt respectively, U1 and U7smOPT snRNAs are comparatively small and easily encodable in a variety of genetic delivery vehicles, from lipid nanoparticles to adeno-associated virus.

### A>I editing with engineered U snRNAs

**Fig. 1:**
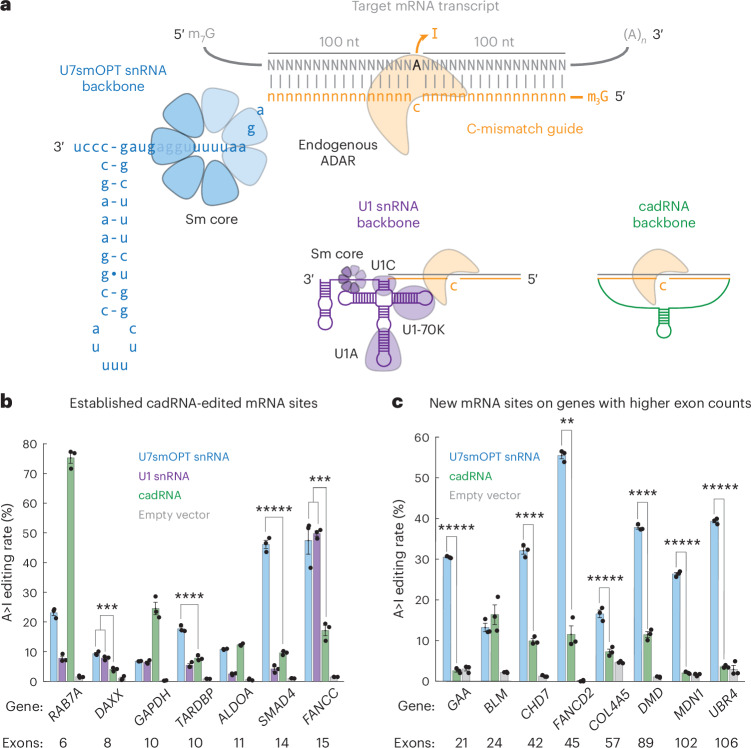
Programmable U snRNAs edit A>I on endogenous human transcripts with stronger activity on target genes containing high exon counts. **a**, Schematic of three different RNA-guided A>I base editors targeting an mRNA transcript with the same C-mismatch guide: U7smOPT (blue), U1 snRNA (purple) and cadRNA (green). **b**, Editing percent performance by transfection in HEK293T cells of three A>I base editors on target sites from various human genes. A>I overperformance significance versus cadRNA: ***P < 0.001, ****P < 0.0001 and *****P < 0.00001 (one-way analysis of variance (ANOVA), Bonferroni correction for multiple comparisons) (*DAXX* U7:cad, 1 × 10^−4^; *DAXX* U1:cad, 9 × 10^−4^; *TARDBP* U7:cad, 5 × 10^−5^; *SMAD4* U7:cad, 4 × 10^−7^; *FANCC* U7:cad, 9 × 10^−4^; *FANCC* U1:cad, 6 × 10^−4^). Error bars reflect the s.e.m. (*n* = 3 biological replicates per condition). **c**, Editing percentage performance by transfection in HEK293T cells of U7smOPT snRNA and cadRNA A>I base editors on target sites from human genes with high exon counts. A>I overperformance significance versus cadRNA: **P < 0.01, ****P < 0.0001 and *****P < 0.00001 (one-way ANOVA) (*GAA*, 2 × 10^−7^; *CHD7*, 4 × 10^−5^; *FANCD2*, 0.0012; *COL4A5*, 7 × 10^−6^; *DMD*, 3 × 10^−5^; *MDN1*, 5 × 10^−7^; *UBR4*, 1 × 10^−7^). Error bars reflect the s.e.m. (*n* = 3 biological replicates per condition).

Of the existing single-component A>I programmable guided RNA scaffolds, circularized ADAR-recruiting RNAs (cadRNAs) have demonstrated potent editing using a simple design^10,11^. With their elegant circularization by autoligating twister ribozymes, cadRNAs effectively withstand degradation by exoribonucleases to sustain strong expression in cells. cadRNAs contain a C-mismatch guide with typically 100-nt homology regions flanking either side of the mismatched C and occasionally mismatches and loops throughout these flanking regions to inhibit spurious bystander editing by ADAR. Because of their shared nuclear localization with ADAR, U snRNAs may be more efficient A>I editors than cadRNAs. Moreover, spliceosomal component Sm proteins have been found to associate with ADAR1 and ADAR2 (ref. ^22^). To test this conjecture, we replaced the cadRNA backbone (in a U6 promoter and U6 terminator cassette) with either the U7smOPT snRNA backbone (in a U7 promoter and U7 terminator cassette) or U1 snRNA backbone (in a U1 promoter and U1 terminator cassette) at the 3′ end of fixed C-mismatch guides (Fig. 1a).

**Extended Data Fig. 1:**
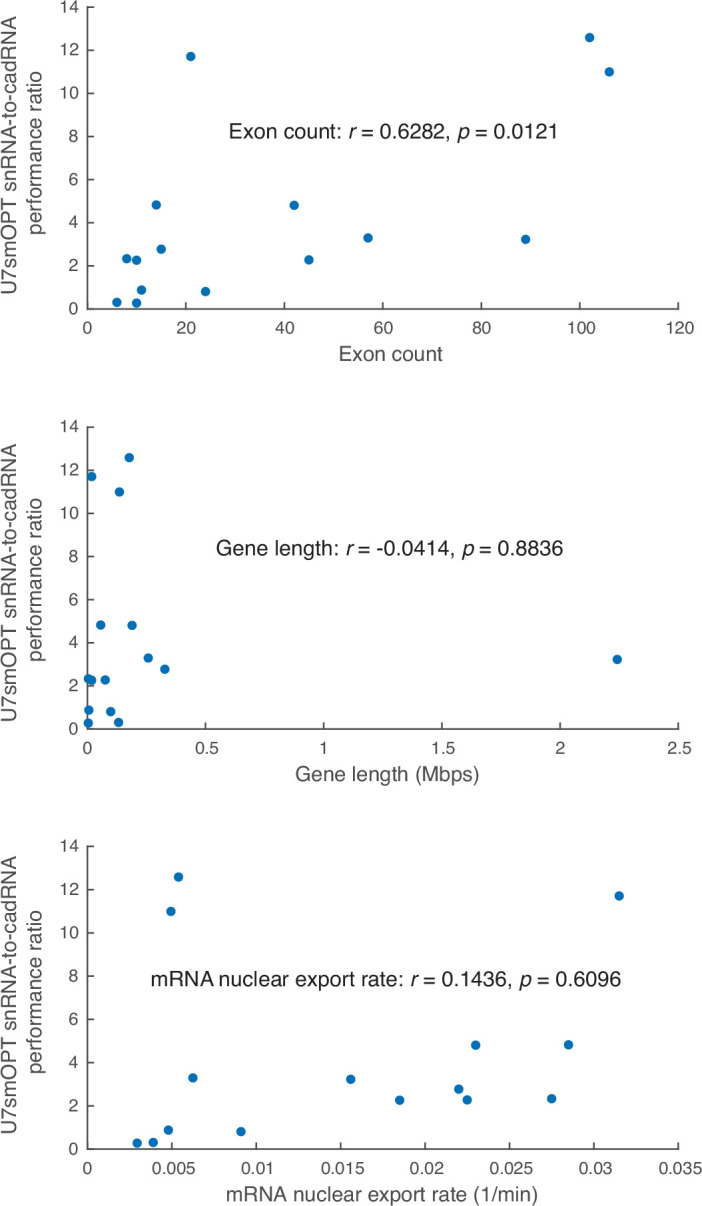
Competing model test for U7smOPT snRNA-to-cadRNA performance ratio. Scatter plots with corresponding Pearson correlation coefficients (*r*) and *p-*values for three competing models of U7smOPT snRNA-to-cadRNA performance ratio: gene exon counts, gene lengths and empirical gene mRNA nuclear export rates. n = 15 genes (all targets from Fig. 1b, c).

A head-to-head A>I editing test of the two U snRNAs against cadRNA across seven previously published endogenous loci and associated C-mismatch guide sequences in HEK293T cells revealed several findings (Fig. 1b)^10^. Firstly, U1 snRNA almost invariably performed more poorly than U7smOPT snRNA. We reasoned that its greater molecular complexity and proclivity for splicing machinery recruitment caused this limiting effect; thus, we disregarded U1 snRNA as a construct for the remainder of our study. Secondly, although U7smOPT snRNA bested cadRNA across four of the seven loci, neither initially appeared a clear winner. Lastly, U7smOPT snRNA most unambiguously outshone cadRNA editing performance at loci on *SMAD4* and *FANCC*, genes with the highest exon counts. We, therefore, hypothesized that the relative A>I editing performance of U7smOPT snRNA compared to cadRNA across target genes correlates positively with gene exon count. To test this theory further, we compared U7smOPT snRNA and cadRNA performance on eight new loci of genes with progressively higher exon counts (Fig. 1c). On all target genes except *BLM*, U7smOPT snRNA convincingly outperformed cadRNA across high exon count gene loci. Lending more credence to our theory, the ratio of U7smOPT snRNA-to-cadRNA editing efficiency over the 15 tested genes correlated moderately and statistically significantly with exon count (Pearson correlation coefficient *r* = 0.6282, *P* = 0.0121) in contrast with unsupported alternative hypotheses of gene length (*r* = −0.0414, *P* = 0.8836) or mRNA nuclear export rate as reported in a recent study (*r* = 0.1436, *P* = 0.6096) (Extended Data Fig. 1)^23^. Given that genes with high exon count tend to be larger and more prone to accumulating disease-relevant mutations (as is the case for *DMD*, in which ~15% of Duchenne muscular dystrophy-implicated mutations are nonsense)^24^, U7smOPT snRNAs present an attractive new modality for treating PTC diseases.

### Off-target genetic perturbations of A>I snRNAs

**Fig. 2:**
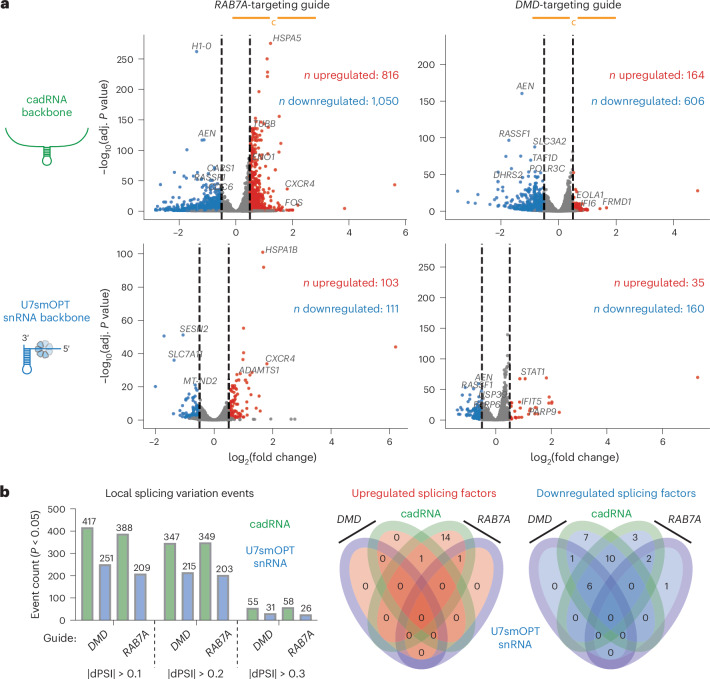
A>I snRNAs perturb fewer genes than cadRNAs. **a**, Scatter plots of differential gene expression analysis against empty control (pUC19) of cadRNA backbone versus U7smOPT snRNA backbone for *RAB7A*-targeting and *DMD*-targeting guides. Cutoffs for significance: |log_2_(fold change)| > 0.5 and adjusted *P* value < 0.05. Upregulated genes are colored in red and downregulated genes are colored in blue (*n* = 2 biological replicates per condition). **b**, Left: number of LSV events from differential splicing analysis against empty control (pUC19) of cadRNA backbone versus U7smOPT snRNA backbone for *RAB7A*-targeting and *DMD*-targeting guides, with *P* < 0.05 and different thresholds of dPSI for events. Right: four-way Venn diagrams of the number of significantly upregulated and downregulated splicing factors represented in the significantly perturbed genes from **a** (*n* = 2 biological replicates per condition).

**Extended Data Fig. 2:**
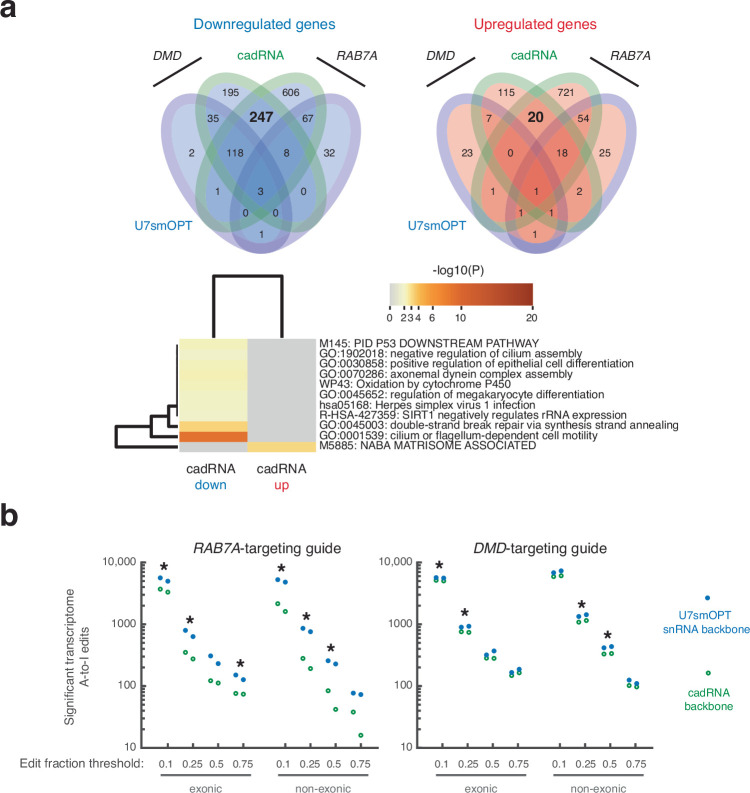
Significant gene perturbations by RNA-guided A > I base editors. **a**, 4-way Venn diagrams of the number of significantly downregulated and upregulated genes across conditions from Fig. 2 (top). Enriched pathway heatmap of downregulated and upregulated genes conserved across both cadRNA guides (bottom). **b**, Counts of significant transcriptome A > I edits, both exonic and non-exonic, absent in both empty control (pUC19) condition replicates of cadRNA backbone versus U7smOPT snRNA backbone for *RAB7A*- and *DMD*-targeting guides, with different edit fraction thresholds (1 = 100% editing). Edit count significance of U7smOPT versus cadRNA: *p* < 5e-2 (one-way ANOVA) (*RAB7A* exonic 0.1, 0.25, 0.75: 0.0422, 0.0476, 0.0336; *RAB7A* non-exonic 0.1, 0.25, 0.5: 0.0124, 0.0134, 0.0196; *DMD* exonic 0.1, 0.25: 0.0179, 0.0107; *DMD* non-exonic 0.25, 0.5: 0.0427, 0.0124). n = 2 biological replicates per condition.

Next, we asked how U7smOPT snRNAs compared to cadRNAs with respect to off-target genetic perturbations. Selecting one guide for which cadRNA outperformed (*RAB7A*-targeting) and one for which U7smOPT snRNA outperformed (*DMD*-targeting), we conducted differential gene expression analysis with DESeq2 on two replicates of RNA sequencing (RNA-seq) data from each condition compared to empty vector (significance cutoffs of |log_2_(fold change)| > 0.5 and adjusted *P* value < 0.05) (Fig. 2a, Supplementary Fig. 1 and Supplementary Dataset 1)^25^. In analyzing the data, we removed apparent overexpression of *DMD* because of a library preparation artifact, as applied in previous work (Supplementary Fig. 2)^10^. In either case, U7smOPT snRNA produced far fewer genetic perturbations (~4–8-fold) than cadRNA, in both upregulated and downregulated genes. Notably, more misregulated genes are shared exclusively between the two cadRNA conditions (267 genes) than between the two *DMD*-targeting conditions (42 genes) or the two *RAB7A*-targeting conditions (121 genes) (Extended Data Fig. 2a). This paradox suggests that guide RNA-independent mechanisms dominate the off-target landscape. Pathway analysis with Metascape of perturbed genes conserved across both cadRNA conditions and absent from either U7smOPT snRNA condition showed notable downregulation of Herpes simplex virus 1 infection and double-stranded break repair by synthesis strand annealing (Extended Data Fig. 2a)^26^. These results imply that structured, stable cadRNAs may be inducing an innate immune response and acting as templates for homologous recombination, either of which would be highly problematic for cells.

While cadRNA may be more genetically perturbative overall, we expected U7smOPT snRNA to generate more splicing changes in the transcriptome. To test this hypothesis, we performed local splicing variation (LSV) analysis with MAJIQ of the RNA-seq datasets (significance cutoff of *P* value < 0.05) (Fig. 2b and Supplementary Fig. 3)^27^. Astonishingly, for both guides across three different thresholds of differential percent spliced in (dPSI), cadRNA produced ~1.5–2-fold more significant LSV events than U7smOPT snRNA. We attribute this unexpected finding not to directly guided splicing perturbations but rather to pleiotropic effects stemming from cadRNA-mediated downregulation of splicing factors (24 versus 7 for *DMD*-targeting and 21 versus 3 for *RAB7A*-targeting, cadRNA versus U7smOPT snRNA, respectively).

Lastly, we examined the number of off-target A>I editing events absent from both empty vector replicates and present across cadRNA and U7smOPT snRNA replicates with our established SAILOR pipeline (significance cutoff: >75% confidence) (Extended Data Fig. 2b)^28^. In the case of each guide, for both exonic and nonexonic edit sites and at various edit fraction thresholds, U7smOPT snRNA generated consistently more transcriptome-wide A>I edits than cadRNA. While concerning, these increased off-target edits do not appear to contribute significantly to transcriptome-wide genetic perturbations (Fig. 2). Moreover, they could be mitigated by reducing the guide length or by introducing mutations to disrupt RNA–RNA hybridization against concerning homology-mediated off-target sites.

### Nuclear RNA base editing with A>I snRNAs

We reasoned that the seeming contradiction between higher cadRNA-mediated genetic perturbations and U7smOPT snRNA-mediated transcriptome-wide A>I edits could be reconciled by known more durable expression of cadRNAs (and accompanying antisense knockdown of transcripts) coupled with higher localization of U7smOPT snRNAs to the ADAR protein-enriched nucleoplasm. Whereas U snRNAs spend most of their life cycle in the nucleoplasm^29^, circular RNAs are actively exported to the cytosol^30^. This localization hypothesis would also explain why A>I snRNA (C-mismatch guide with U7smOPT snRNA backbone) outperforms cadRNA on high exon count gene mRNAs, which typically persist longer in the nucleus because of more extensive splicing before nuclear export.

**Fig. 3:**
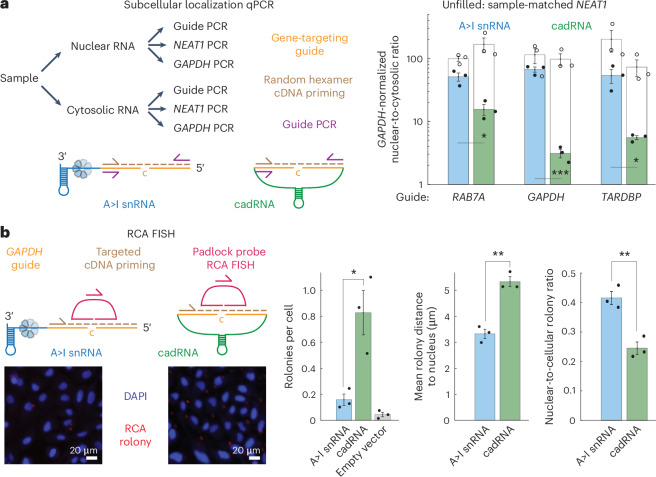
A>I snRNAs localize more persistently to the nucleus. **a**, Left: schematic of subcellular localization qPCR to determine nuclear-to-cytosolic enrichment of A>I snRNA and cadRNA A>I base editors. Right: results from subcellular localization qPCR of the A>I base editors with guides targeting three different genes, using the lncRNA *NEAT1* as a positive control for the assay. Nuclear-to-cytosolic enrichment significance versus cadRNA: *P < 0.05 and ***P < 0.001 (one-way ANOVA) (*RAB7A*, 0.0121; *GAPDH*, 5 × 10^−4^; *TARDBP*, 0.0216). Error bars reflect the s.e.m. (*n* = 3 biological replicates per condition). **b**, RCA FISH schematic, representative images and plots of rolonies per cell, mean rolony distance to nucleus and nuclear-to-cellular rolony ratio for *GAPDH*-targeting A>I snRNA and cadRNA. RCA FISH was performed in human U-2 OS cells with ×10 magnification images. Images depicting fluorescence signal are small representations of the total number of nuclei (thousands per biological replicate) and are not suitable for visual interpretation. Pixel resolution: 752 nm × 752 nm. Significance of difference: *P < 0.05 and **P < 0.01 (one-way ANOVA) (rolonies per cell, 0.0191; mean rolony distance to nucleus, 0.0016; nuclear-to-cellular rolony ratio, 0.0051). Error bars reflect the s.e.m. (*n* = 3 biological replicates per condition).

To test the localization hypothesis, we devised a subcellular localization qPCR assay, whereby qPCR performed on both A>I scaffolded guides and genes from nuclear and cytosolic fractionated RNA enables inferred nuclear-to-cytosolic ratio comparison between A>I snRNA and cadRNA for equivalent guides (Fig. 3a). As expected, across three different guides, A>I snRNA localized more highly to the nucleus than cadRNA, with a sample-matched *NEAT1* positive control showing no significant difference in nuclear-to-cytosolic ratio between conditions (Fig. 3a). We orthogonally validated the enriched nuclear localization of A>I snRNA using single-molecule rolling circle amplification fluorescence in situ hybridization (RCA FISH), demonstrating that A>I snRNAs reside ~2 μm closer to the nucleus on average and are ~70% more localized (~42% versus ~25%) within the nucleus than cadRNAs (Fig. 3b). Additionally, RCA FISH enabled us to quantify the expression of cadRNA relative to A>I snRNA, which is ~5-fold for the *GAPDH* guide and likely impacts relative A>I editing performance. Unlike the subcellular localization qPCR experiment in which cadRNA qPCR threshold cycles for nuclear and cytosolic fractions cancel each other out, cadRNA and A>I snRNA qPCR threshold cycles cannot be directly compared to each other because of differences in circularized and linear templated strand-displacing complementary DNA (cDNA) synthesis.

**Fig. 4:**
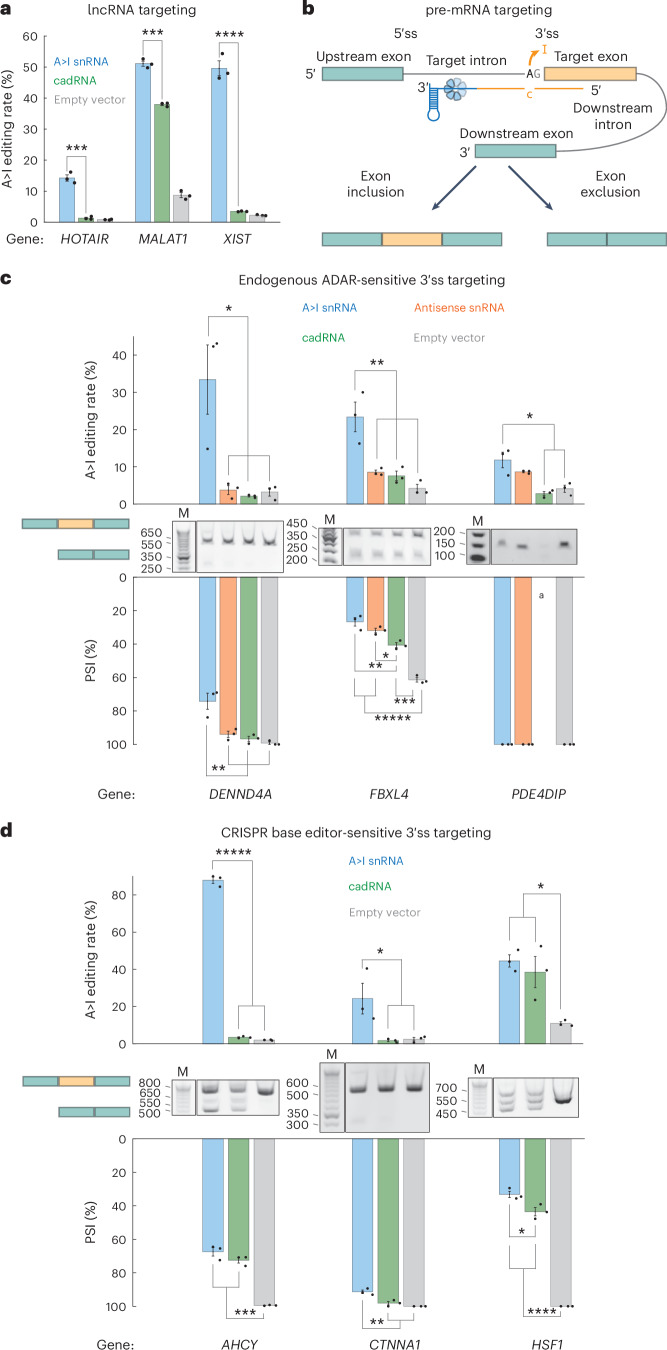
A>I snRNAs edit lncRNAs and pre-mRNAs more efficiently. **a**, Editing percentage performance by transfection in HEK293T cells of A>I snRNA and cadRNA A>I base editors on lncRNAs. A>I overperformance significance versus cadRNA: ***P < 0.001 and ****P < 0.0001 (one-way ANOVA) (*HOTAIR*, 3 × 10^−4^; *MALAT1*, 1 × 10^−4^; *XIST*, 4 × 10^−5^). Error bars reflect the s.e.m. (*n* = 3 biological replicates per condition). **b**, Schematic of A>I snRNA targeting 3′ splice site of pre-mRNA. **c**,**d**, A>I editing, RT–PCR gels and quantified PSI performance by transfection in HEK293T cells of A>I snRNAs, antisense snRNAs, cadRNAs and empty control (pUC19) for endogenous ADAR-sensitive (**c**) and CRISPR base editor-sensitive (**d**) 3′ splice sites of three human genes. Significance of differences: *P < 0.05, **P < 0.01, ***P < 0.001, ****P < 0.0001 and *****P < 0.00001 (one-way ANOVA, Bonferroni correction for multiple comparisons) (*DENND4A* editing A>I:anti, A>I:cad and A>I:empty, 0.0130, 0.0094 and 0.0117; *FBXL4* editing A>I:anti, A>I:cad and A>I:empty: 0.0078, 0.0052 and 0.0015; *PDE4DIP* editing A>I:cad and A>I:empty, 0.0040 and 0.0104; *DENND4A* splicing A>I:anti, A>I:cad and A>I:empty, 0.0055, 0.0024 and 0.0012; *FBXL4* splicing A>I:cad, anti:cad, A>I:empty, anti:empty and cad:empty, 0.0030, 0.0480, 1 × 10^−5^, 1 × 10^−5^ and 2 × 10^−4^; *AHCY* editing A>I:cad and A>I:empty, 5 × 10^−9^ and 5 × 10^−9^; *CTNNA1* editing A>I:cad and A>I:empty, 0.0474 and 0.0498; *HSF1* editing A>I:empty and cad:empty, 0.0116 and 0.0289; *AHCY* splicing A>I:empty and cad:empty, 1 × 10^−5^ and 1 × 10^−4^; *CTNNA1* splicing A>I:cad and A>I:empty, 0.0046 and 0.0013; *HSF1* splicing A>I:cad, A>I:empty and cad:empty, 0.0209, 1 × 10^−5^ and 1 × 10^−5^). Error bars reflect the s.e.m. (*n* = 3 biological replicates per condition). ^a^Gel quantification not reliable because of low abundance of dominant band. Source data

**Extended Data Fig. 3:**
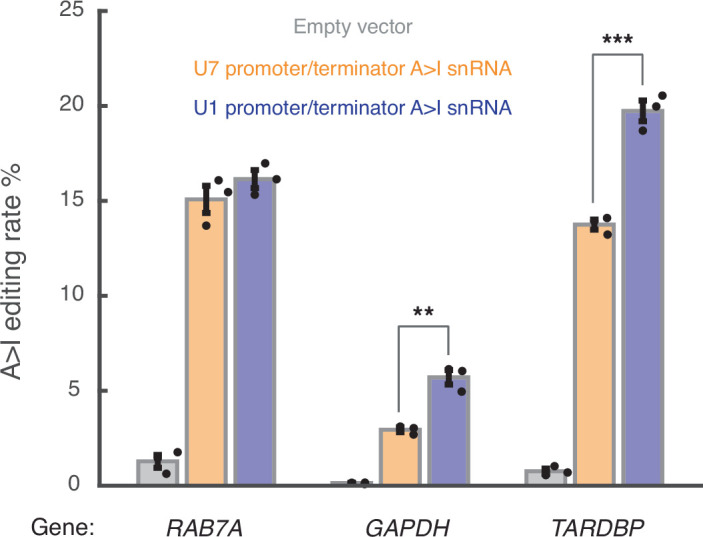
Editing performance of A > I snRNAs with U7 versus U1 snRNA promoter/terminator cassette. Editing percent performance by transfection in HEK293T cells of A > I snRNAs targeting three different genes and driven by either U7 or U1 snRNA promoter/terminator cassette. Overperformance significance versus U7 promoter: **, ***: *p* < 1e-2, 1e-3 (one-way ANOVA) (*GAPDH* 0.0023, *TARDBP* 6e-4). Error bars reflect standard error of mean. n = 3 biological replicates per condition.

Given this exciting nuclear localization discovery, we wondered whether A>I snRNAs could be leveraged to edit long noncoding RNAs (lncRNAs) and pre-mRNAs, applications not robustly demonstrated with single-component A>I programmable guided RNA scaffold modalities. To boost editing activity in these subsequent experiments, we engineered U7smOPT scaffold A>I snRNAs within a U1 promoter and U1 terminator cassette for more efficient construct expression (Extended Data Fig. 3). We first targeted three well-characterized lncRNAs: *HOTAIR*, *MALAT1* and *XIST* (Fig. 4a). As predicted, A>I snRNA outperformed cadRNA in all cases. Next, we targeted pre-mRNA 3′ splice sites, whose disruption leads to exon exclusion (Fig. 4b). In addition to testing A>I snRNA and cadRNA, we included an antisense snRNA condition (C-mismatch eliminated from guide) to control for the effects of spliceosomal assembly steric hindrance. We first tested three 3′ splice site contexts for which native A>I editing has previously been implicated in splicing perturbation through ADAR knockdown: *DENND4A*, *FBXL4* and *PDE4DIP* (Fig. 4c and Supplementary Fig. 4)^31^. A>I snRNAs edited all three pre-mRNA loci more efficiently than the other conditions, with editing rates ranging from ~10% to 30%. These increased editing rates modestly translated to improved exon skipping of A>I snRNA over both cadRNA and antisense snRNA (*DENND4A* –dPSI, ~25% A>I snRNA versus ~5% antisense snRNA; *FBXL4* –dPSI, ~35% A>I snRNA versus ~29% antisense snRNA), except for *PDE4DIP*, in which case splicing changes fell below the sensitivity of the reverse transcription (RT)–PCR assay and a weakly editing cadRNA degraded the transcript. Lastly, we tested three additional 3′ splice site contexts for which CRISPR–Cas9 adenine deaminase base editing of DNA results in exon skipping (Fig. 4d and Supplementary Fig. 5)^32^. Again, A>I snRNA outperformed cadRNA in editing efficiency (~20–85%) and exon skipping (~10–65%). These splicing results indicate a new use of A>I snRNAs whose efficacy above antisense-mediated steric hindrance of spliceosomal assembly may depend largely on *cis*-splicing factors.

### Increased pseudouridylation efficiency with U>Ψ snRNAs

**Fig. 5:**
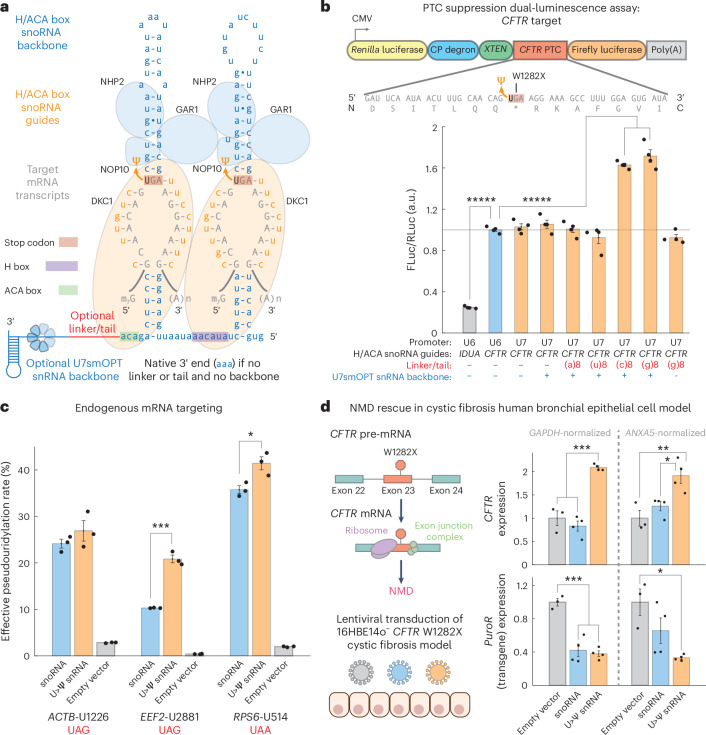
U>Ψ snRNAs demonstrate increased potency over engineered snoRNAs in pseudouridylation and PTC suppression. **a**, Design of engineered U>Ψ snRNA, with guided H/ACA box snoRNA targeting an mRNA transcript fused by RNA linker to U7smOPT backbone. **b**, Top: schematic of dual-luciferase reporter to quantify PTC suppression of *CFTR*^W1282X^ mutant by tested U>Ψ snRNA designs. Bottom: luciferase ratio performance by cotransfection in HEK293T cells of various U>Ψ snRNA designs on PTC suppression dual-luciferase reporter. FLuc/RLuc significance versus U6 promoter-driven CFTR guide H/ACA box snoRNA without linker or tail and U7smOPT backbone: *****P < 0.00001 (one-way ANOVA, Bonferroni correction for multiple comparisons) (snoRNA *IDUA*:*CFTR*, 1 × 10^−12^; *CFTR* snoRNA:(c)8, 7 × 10^−11^; *CFTR* snoRNA:(g)8, 3 × 10^−12^). Error bars reflect the s.e.m. (*n* = 4 biological replicates per condition). **c**, Effective pseudouridylation performance by transfection in HEK293T cells of guided U>Ψ snRNAs versus H/ACA box snoRNAs on stop codon context sequences from three human genes. Pseudouridylation overperformance significance versus snoRNA: *P < 0.05 and ***P < 0.001 (one-way ANOVA) (*EEF2*, 2 × 10^−4^; *RPS6*, 0.0264). Error bars reflect the s.e.m. (*n* = 3 biological replicates per condition). **d**, Left: schematic of *CFTR*^W1282X^-induced NMD and 16HBE14o^−^ lentiviral transduction experiment. Right: *CFTR* and *PuroR* (transgene) expression evaluated by qPCR, with both *GAPDH* and *ANXA5* as housekeeping genes. Significance of expression difference: *P < 0.05, **P < 0.01 and ***P < 0.001 (one-way ANOVA, Bonferroni correction for multiple comparisons) (*CFTR*-*GAPDH* empty:U>Ψ, 2 × 10^−4^; *CFTR*-*GAPDH* snoRNA:U>Ψ, 1 × 10^−5^; *CFTR*-*ANXA5* empty:U>Ψ, 0.0082; *CFTR*-*ANXA5* snoRNA:U>Ψ, 0.0328; *PuroR*-*GAPDH* empty:snoRNA, 3 × 10^−4^; *PuroR*-*GAPDH* empty:U>Ψ, 2 × 10^−4^; *PuroR*-*ANXA5* empty:U>Ψ, 0.0173). Error bars reflect the s.e.m. (*n* = 3 biological replicates for empty vector condition; *n* = 4 biological replicates for snoRNA and U>Ψ snRNA conditions).

Given our success in localizing A>I snRNAs to the nucleus for enhanced A>I editing, we applied a similar approach to U>Ψ RNA base editing. H/ACA box snoRNAs, which catalyze U>Ψ modification (editing) of rRNAs and snRNAs, localize predominantly to the nucleolus. We hypothesized that more nucleoplasmic localization of programmable guided H/ACA box snoRNAs through fusion to a U7smOPT snRNA backbone would direct the snoRNAs away from the nucleolus for more efficient U>Ψ editing on coding RNAs (Fig. 5a).

**Extended Data Fig. 4:**
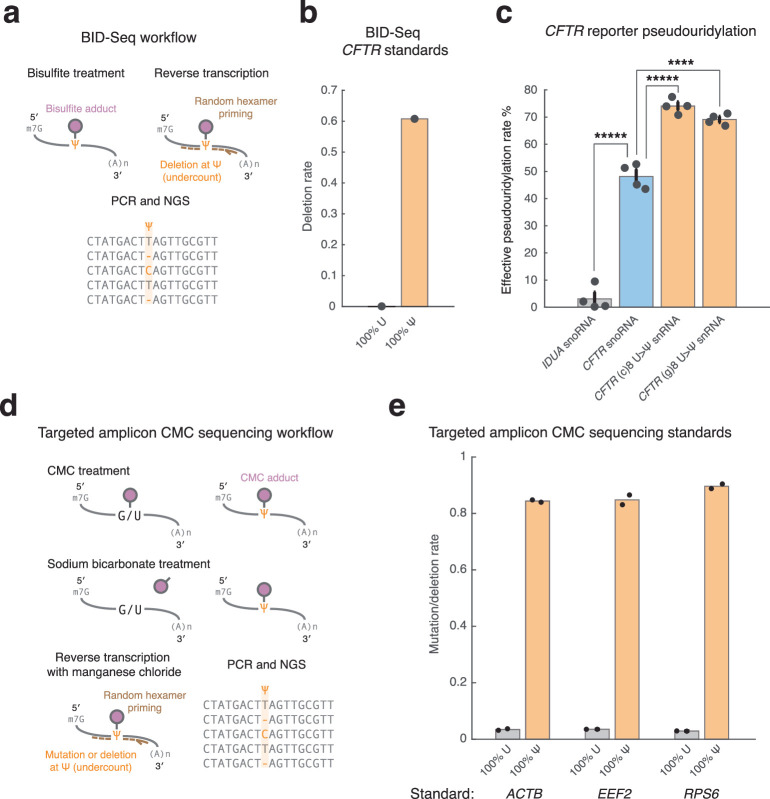
Targeted amplicon sequencing for evaluating endogenous targeted pseudouridylation. **a**, Schematic of BID-Seq to infer pseudouridylation of endogenous targeted mRNA by deletion rate. **b**, BID-Seq deletion rates on synthetic RNA standards (100% U and 100% Ψ at targeted base) for *CFTR* reporter locus. n = 1 technical replicate per condition. **c**, Effective pseudouridylation performance by transfection in HEK293T cells of guided U > Ψ snRNAs with (g)8 and (c)8 linkers versus H/ACA box snoRNA on *CFTR* reporter. Pseudouridylation difference significance versus snoRNA: ****, *****: *p* < 1e-4, 1e-5 (one-way ANOVA, Bonferroni correction for multiple comparisons) (snoRNA *IDUA*:*CFTR* 3e-9, *CFTR* snoRNA:(c)8 1e-5, *CFTR* snoRNA:(g)8 2e-6). Error bars reflect standard error of mean. n = 4 biological replicates per condition. **d**, Schematic of targeted amplicon CMC sequencing to infer pseudouridylation of endogenous targeted mRNA by mutation/deletion rate. **e**, Targeted amplicon CMC sequencing mutation/deletion rates on synthetic RNA standards (100% U and 100% Ψ at targeted base) for endogenous *ACTB*, *EEF2*, and *RPS6* loci. n = 2 technical replicates per condition.

To test whether an snRNA approach could enhance U>Ψ editing, we designed a monocistronic, internally controlled dual-luminescence reporter harboring a cystic fibrosis-implicated PTC from human *CFTR*^W1282X^ between *Renilla* luciferase (RLuc) and firefly luciferase (FLuc) (Fig. 5b). In a cotransfection experiment in HEK293T cells, expression of an established *CFTR* target-guided H/ACA box snoRNA increased the FLuc/RLuc luminescence ratio ~4-fold over a negative control *IDUA* target-guided H/ACA box snoRNA, validating the assay’s sensitivity as a proxy for PTC readthrough. Of all linkers tested between *CFTR* target-guided H/ACA box snoRNA and U7smOPT snRNA backbone, both the (c)8 and (g)8 linkers (5′-cccccccc-3′ and 5′-gggggggg-3′) resulted in significant FLuc/RLuc luminescence ratio increases, with the (g)8 linker raising the luminescence ratio by ~70% above the snoRNA condition, whereas a (g)8 tail without U7smOPT snRNA backbone had no significant effect. Pseudouridylation quantitation by bisulfite sequencing (BID-seq) revealed a concomitant U>Ψ editing rate increase of ~40% for (c)8 and (g)8 linker conditions (~70% editing) compared to the snoRNA condition (~50% editing), indicating a superlinear relationship between protein-level PTC readthrough and pseudouridylation rates (Extended Data Fig. 4a–c)^33^. Meanwhile, neither the (a)8 nor the (u)8 linkers (5′-aaaaaaaa-3′ and 5′-uuuuuuuu-3′) increased the luminescence ratio. Considering these results, we suspect that, unlike the other linkers tested, the (c)8 and (g)8 linkers help stabilize the expanded RNA scaffold. Potentially, the (c)8 and (g)8 linkers, when followed by U7smOPT snRNA backbone, form an endoribonuclease-resistant secondary structure that prevents 3′-end processing of the H/ACA box snoRNA. On the other hand, the (a)8 and (u)8 linkers may recruit poly(A)-binding and poly(U)-binding proteins, respectively, that destabilize the construct.

We next tested the H/ACA box snoRNA–(g)8 linker–U7smOPT snRNA backbone fusions (U>Ψ snRNAs) on three endogenous loci in HEK293T cells and quantified the U>Ψ editing rate using targeted amplicon CMC sequencing (Fig. 5c and Extended Data Fig. 4d,e)^16^. On all three loci, two with statistical significance and one >2-fold, U>Ψ snRNAs outperformed H/ACA box snoRNAs as predicted (pseudouridylation rates of ~20–40%). This generalization suggests that increased RNA-guided targeted pseudouridylation can be achieved without cytoplasmic DKC1 overexpression, a reportedly successful strategy that nevertheless poses the risk of promoting cancer progression^16^.

**Extended Data Fig. 5:**
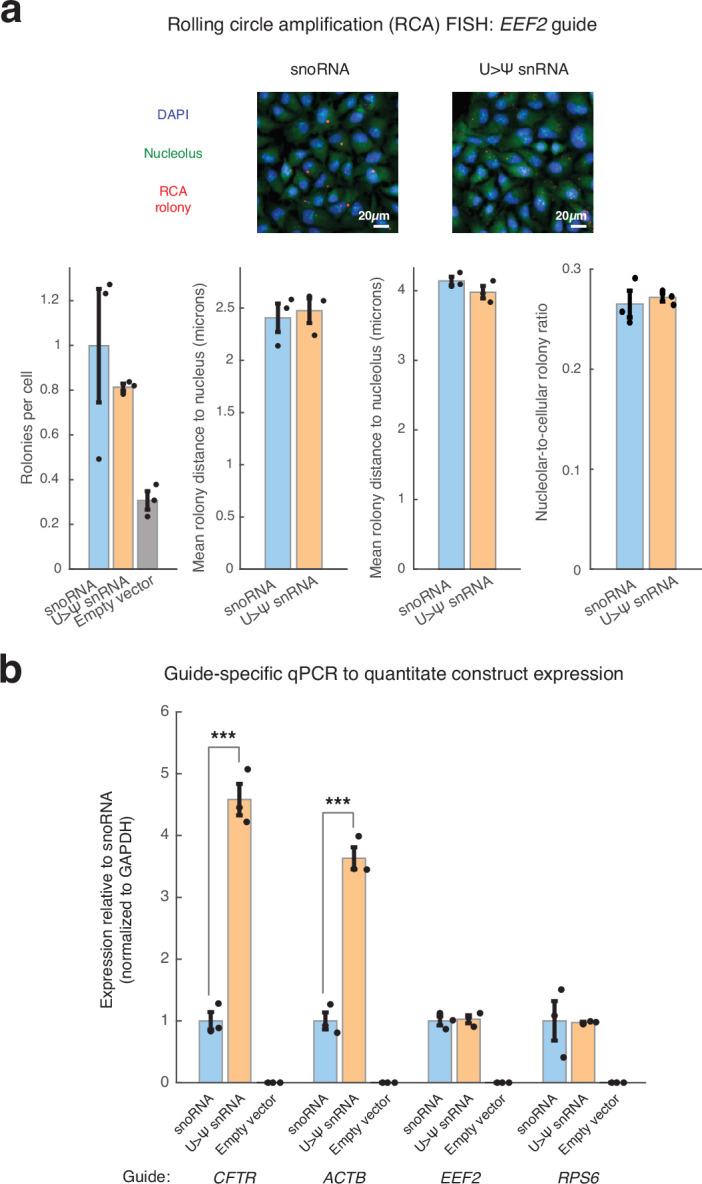
Experiments to evaluate mechanism of enhanced pseudouridylation by U > Ψ snRNAs. **a**, Rolling circle amplification FISH (RCA FISH) schematic, representative images and plots of rolonies per cell, mean rolony distance to nucleus/nucleolus and nucleolar-to-cellular rolony ratio for *EEF2*-targeting H/ACA box snoRNA and U > Ψ snRNAs. RCA FISH was performed in human U-2 OS cells with ×10 magnification images. Images depicting fluorescent signal are small representations of total number of nuclei (thousands per biological replicate) and are not suitable for visual interpretation. Scale bar: 20 microns. Pixel resolution: 752 nm x 752 nm. **b**, Guide-specific qPCR to quantitate expression levels of H/ACA box snoRNA and U > Ψ snRNA constructs targeting *CFTR*, *ACTB*, *EEF2* and *RPS6*. Significance of difference: ***: *p* < 1e-3 (one-way ANOVA) (*CFTR* 3e-4, *ACTB* 3e-4). Error bars reflect standard error of mean. n = 3 biological replicates per condition.

Given that U>Ψ snRNAs reproducibly enhance pseudouridylation over H/ACA box snoRNAs, we sought to assess our working hypothesis of snRNA-mediated subcellular localization into the nucleoplasm and out of the nucleolus. However, RCA FISH of the *EEF2* guide on both U>Ψ editing RNA scaffolds did not conclusively demonstrate either increased nuclear localization or decreased nucleolar localization of the U>Ψ snRNA compared to the H/ACA box snoRNA (Extended Data Fig. 5a). Notwithstanding these results, it is conceivable that snRNAs may localize the constructs to other subnuclear structures such as nuclear speckles, where DKC1 has been found to reside^34^. As an alternative hypothesis, U>Ψ snRNAs may achieve higher cellular abundance through Sm core-mediated stabilization and, thus, greater construct-to-target stoichiometry. Guide-specific qPCR revealed this hypothesis to be the case for both *CFTR*-targeting and *ACTB*-targeting constructs but, importantly, for neither *EEF2*-targeting nor *RPS6*-targeting constructs (Extended Data Fig. 5b). Although we have not totally resolved the molecular mechanism of U>Ψ snRNA efficiency, we posit that the snRNA enhances pseudouridylation activity through a balance of both greater construct stability and presently undetermined subnuclear localization and effector recruitment.

Lastly, we tested the ability of the U>Ψ snRNA to improve NMD rescue in a human bronchial epithelial cystic fibrosis model (Fig. 5d). PTCs lead to NMD through the downstream presence of exon junction complexes, and PTC suppression rescues PTC-harboring mRNAs from NMD. We transduced 16HBE14o^−^ cells harboring the *CFTR*^W1282X^ mutation with lentivirus encoding empty vector, *CFTR*-targeting snoRNA or previously optimized *CFTR*-targeting U>Ψ snRNA construct (Fig. 5a). Treatment with *CFTR*-targeting U>Ψ snRNA increased *CFTR* expression ~2-fold when evaluated by qPCR against two distinct housekeeping genes (*GAPDH* and *ANXA5*). However, treatment with *CFTR*-targeting snoRNA at an equivalent transgene expression level did not achieve discernible NMD rescue. Encouragingly, this enhanced activity of U>Ψ snRNAs will enable lower dosage and, thus, safer therapeutics for the same PTC suppression efficacy.

## Discussion

RNA base editing by programmable single-component guided RNA scaffolds has demonstrated promise as both a minimally invasive and a target-specific approach to gene editing. In this study, we engineered U snRNAs to enhance such systems for A>I and U>Ψ editing on mammalian transcripts. In either base editing case, snRNAs improved system safety and/or efficacy performance over state-of-the-art approaches, with an aspiration toward preclinical targeted suppression of PTC diseases. Given that Sm core proteins are highly conserved and expressed in all mammalian cells, we expect our findings to translate effectively to other cell types and broadly to PTC disease therapeutics.

Excitingly, we showed in our study that U>Ψ snRNA significantly increases PTC readthrough over state-of-the-art engineered snoRNA in a human cellular disease model of cystic fibrosis. Given the generalizability of enhanced pseudouridylation to other endogenous mRNA targets and an observed superlinear relationship between protein-level PTC readthrough and pseudouridylation rates, U>Ψ snRNAs likely could broadly advance the development of PTC disease gene therapies. Beyond genetically encoded gene therapies, U>Ψ snRNAs could also be administered to patients through nonviral delivery methods such as lipid nanoparticles. Here, the snRNA backbone may further aid in nuclear delivery, as snRNAs spend part of their life cycle at the cytoplasmic SMN complex before returning to the nucleus^35^.

More efficient genetically encodable single-component RNA-guided editing of RNA noncoding regions, including the 3′ splice sites of pre-mRNA, enables other therapeutic opportunities. For example, RNA base editor snRNAs could edit intronic RNA-binding protein (RBP) motifs to displace destabilizing RBPs and increase nuclear RNA expression. When coupled with single-component RNA-guided translational activation systems, RNA base editor snRNAs could provide an additional boost to protein expression^36^.

Importantly, snRNA enhancements to RNA base editing systems are guide independent, suggesting an approach that will benefit researchers even as they orthogonally optimize RNA guides for on-target base editing efficiency and specificity^37^. While our study focused on the U7smOPT snRNA backbone, there are likely further optimizations to be made to the sequence of the snRNA enhancement for augmented scaffold stability and nucleoplasmic RNA localization^38^. In addition to the H/ACA box snoRNA, we anticipate that snRNA components could enhance programmable base editing by other noncoding RNAs, such as C/D box snoRNAs^39–42^. Future studies will undoubtedly explore these open questions and potential use cases.

## Methods

### Cloning of plasmids

gRNA and snoRNA plasmids (Supplementary Tables 1 and 2) were subcloned into pUC19 (N3041S, New England Biolabs) and pcDNA 3.1(-) (V79520, Life Technologies Corporation) using EcoRI-HF and BamHI-HF digestion followed by Gibson assembly (E2611L, New England Biolabs). Lentiviral plasmids, containing CFTR-targeting small RNAs, were ordered as gene fragments (Twist) and similarly assembled into a lentiviral transfer vector with an Ef1a core-eGFP-PuroR cassette (Supplementary Table 1) by Gibson assembly. Nonlentiviral Gibson assemblies were transformed into Mix & Go! competent cells JM109 (T3005, Zymo Research) and plated on Luria–Bertani (LB) agar with antibiotic. Lentiviral Gibson assemblies used One Shot Stbl3 chemically competent *Escherichia coli* (C737303, Thermo Fisher Scientific). All plasmids were cultured in LB medium with antibiotic, miniprepped using a QIAprep Spin miniprep kit and verified by sequencing and SnapGene analysis.

### Cell culture

Human HEK 293T cells (632180, Takara Bio) and U-2 OS cells (HTB-96, American Type Culture Collection) were maintained in D10 (DMEM (4.5 g L^−1^ d-glucose) supplemented with 10% FBS (Gibco) and 1% penicillin–streptomycin (10,000 U per ml) (Gibco) at 37 °C with 5% CO_2_. Cells were periodically passaged once at 70–90% confluency by dissociating with TrypLE Express enzyme (Gibco) at a ratio of 1:10.

The 16HBE14o^−^ *CFTR*^W1282X^ human bronchial epithelial cells (Cystic Fibrosis Foundation) were cultured at 37 °C, 5% CO_2_ in 16HBE14o^−^ expansion medium (α-MEM (Sigma-Aldrich, M2279-500ML) supplemented with 10% FBS (Gibco), 1% GlutaMax (ThermoFisher Scientific) and 1% penicillin–streptomycin (10,000 U per ml) (Gibco)). Before cell plating, flasks were coated for ~2 h with an unrinsed fibronectin and collagen ECM mixture (97 ml of α-MEM, 2 ml of human fibronectin stock (0.5 mg ml^−1^ in α-MEM; Sigma-Aldrich, F2006-5MG) and 1 ml of PureCol (3 mg ml^−1^ in 0.01 N HCl; Sigma-Aldrich 5006-15MG)). Cells were passaged 1:10 using TrypLE Express when 90–95% confluent. 16HBE14o^−^ *CFTR*^W1282X^ cells were genotyped by harvesting in QuickExtract DNA extraction solution (QE09050, LGC, Biosearch Technologies), followed by PCR with primers containing sequences 5′-GGTCAGGATTGAAAGTGTGCA-3′ and 5′-CTATGAGAAAACTGCACTGGA-3′, PCR purification and amplicon sequencing (Plasmidsaurus).

### Transfections

HEK 293T cells (<30 passages) were transfected using jetOPTIMUS DNA transfection reagent (VWR International, 76299-632). For A>I RNA extractions, cells in 48-well plates were transfected at ~60% confluency with 250 ng of plasmid DNA. For U>Ψ RNA extractions, cells in 12-well plates were transfected at ~60% confluency with 1 µg of plasmid DNA. For luciferase reporter assays, cells in 96-well plates were transfected at ~60% confluency with 100 ng of plasmid DNA (75 ng of guide and 25 ng of reporter). All transfections routinely achieved over 80% efficiency.

### RNA extraction, A>I editing quantification and RNA-seq library preparation

First, 48 h after transfection, cells were washed twice with PBS and RNA was extracted using the Qiagen RNeasy Plus mini kit, eluting in 30 µl. For A>I editing, cDNA was synthesized from 3 µl of RNA in a 10-µl volume using a New England Biolabs ProtoScript II first-strand cDNA synthesis kit and oligo(dT) primers. PCR was performed with 500 nM of specified primers (Supplementary Table 3) using NEBNext Ultra II Q5 master mix, with a 68 °C *T*_m_ and 30-s extension for 30 cycles (mRNA) or 32 cycles (pre-mRNA). PCR products were purified (Qiagen QIAquick) and Sanger-sequenced to quantify A>I editing. RNA-seq libraries were prepared from 1 µg of RNA using the Illumina stranded mRNA prep, ligation kit (20040532, Illumina) following the manufacturer’s protocol. Libraries were sequenced on an Illumina NovaSeq X Plus 10B (100-bp paired-end reads) with a target depth of ~50 million reads per sample.

### Correlation modeling of U7smOPT snRNA versus cadRNA A>I editing

Analysis was performed on the 15 genes targeted in Fig. 1b,c. U7smOPT snRNA-to-cadRNA performance ratios were calculated as the ratios of the mean editing efficiencies for each respective target. Exon counts were taken from GRCh38.p14. Gene lengths were calculated as the difference between start and end base indices of genes from GENCODE version 44 (GRCh38.p14). Mean nuclear export rates of gene mRNAs were calculated as the average of two replicates of k_nucexp_from_nucres.mean in K562 cells from Table S1 of Ietswaart et al.^23^. Pearson’s linear correlation coefficients were taken for the performance ratio versus each hypothesis (exon count, gene length and mean nuclear export rate) across all targets. Data were analyzed and plotted in Matlab (version R2024a).

### RNA-seq alignment

Reads were quality-checked and adaptor-trimmed with FastQC. Paired reads were then aligned to the GENCODE version 44 hg38 primary assembly using STAR aligner (version 2.7.6a). A genome index was built using GRCh38.primary_assembly.genome.fa, gencode.v44.primary_assembly.annotation.gtf and ‘--sjdbOverhang 100’. FASTQ files were mapped with default options and ‘--outSAMtype BAM unsorted’. SAMtools (version 1.3.1) sorted and indexed the resulting BAM files.

### Differential gene expression analysis

Differential expression analysis of RNA-seq reads involved Subread featureCounts (version 1.5.3) for gene quantification and DESeq2 (version 1.39.3) for identifying differentially expressed genes (DEGs). Subread featureCounts generated gene count matrices from paired-end reads, using parameters such as gencode.v44.primary_assembly.annotation.gtf, exon features and filtering for primary alignments, quality score (Q255) and duplicate reads. These matrices were loaded into R and converted to DESeq2 datasets; genes with no expression were removed. DESeq2::DESeq identified DEGs using default parameters, with results saved as .csv files. Volcano plots were generated using RNAlysis 2 (version 3.9.2) with a significance threshold of adjusted *P* < 0.05 and log_2_(fold change) > 0.5. Principal component analysis plots were also created with RNAlysis 2 after normalizing counts by relative log expression and filtering low-expression genes. Gene set enrichment analysis (GSEA) (version 4.3.2) was performed on ranked gene lists, filtered by a log_2_(fold change) threshold of ±0.5. Ranking metrics were calculated as the sum of the product of the log_2_(fold change) and −log_10_(*P* value). GSEA used the c5.go.bp.v2023.2.Hs.symbols.gmt gene set and classic enrichment statistics. DMD was excluded from lists for DMD-targeting guide RNAs because of an artifact. Full DESeq2 outputs are in Supplementary Dataset 1.

### Gene pathway analysis

Significantly upregulated and downregulated genes identified in both cadRNA datasets but not in either U7smOPT snRNA dataset were analyzed for gene pathway significance with Metascape (version 3.5.20240101) using as background all genes without an ‘NA’ adjusted *P* value in all replicates of cadRNA and U7smOPT snRNA datasets from the DESeq2 pipeline.

### Differential splicing analysis

Splicing variations across cadRNA, U7 smOPT and pUC19 controls were assessed using MAJIQ and VOILA (version 2.5). MAJIQ builder created splice graphs and MAJIQ quantifier measured the dPSI of LSVs at known splice sites. VOILA TSV selected genes with *P*(|dPSI| > *x*) > 0.95. PRISM and R were used for plotting.

### Splicing factor analysis

Significantly perturbed splicing factors were identified as a subset of significantly upregulated and downregulated genes from both cadRNA and U7 smOPT datasets contained within the Gene Ontology biological process RNA splicing (GO:0008380)

### Transcriptome-wide A>I editing analysis

Transcriptome-wide RNA editing was quantified using SAILOR (version 1.1.0). Aligned reads were processed to generate base quality MD tags with sAMtools (version 1.3.1) calmd -b and the GENCODE version 44 GRCh38.primary_assembly.genome.fa sequence. Reads with MD tags were analyzed using the SAILOR cwl workflow, using the same reference genome as for alignment and MD tag generation. Edit sites required a SAILOR confidence level exceeding 0.75 for significance. Exonic sites intersected with ‘exon’ features from gencode.v44.primary_assembly.annotation.gtf using ‘BEDTools intersect -s -wa -a’. All other sites were classified as nonexonic. Edit fraction thresholds were applied to SAILOR’s post-pseudocount edit percentage output.

### Subcellular localization qPCR

Cells were washed twice with ice cold PBS and then spun down for 5 min at 300*g*, before aspirating the supernatant. Cells were then resuspended completely by gentle pipetting with 150 μl of buffer A (15 mM Tris-HCl pH 8, 15 mM NaCl, 60 mM KCl, 1 mM EDTA pH 8, 0.5 mM EGTA pH 8, 0.5 mM spermidine and 10 U per ml SUPERase•In RNase Inhibitor (AM2694, Thermo Fisher Scientific)). To this solution, 150 μl of 2× lysis buffer (buffer A with 0.5% NP-40) was added and mixed by inversion. The mixture was incubated for 8 min at 4 °C and then spun down for 5 min at 400*g*. The top 200 μl of supernatant was carefully removed and placed into a new tube (the cytosolic fraction). The remaining supernatant was removed and discarded from the nuclear pellet and this pellet was resuspended in 1 ml of RLN buffer (50 mM Tris-HCl pH 8, 140 mM NaCl, 1.5 mM MgCl_2_, 0.5% NP-40, 10 mM EDTA pH 8, 10 U per ml SUPERase•In RNase inhibitor (AM2694, Thermo Fisher Scientific)). This nuclear resuspension was incubated for 5 min at 4 °C. During the incubation, the cytosolic fraction was spun again for 1 min at 500*g* and its supernatant was collected into a new tube. Next, 500 μl of Trizol LS (10296010, Invitrogen) was added to this cytosolic fraction. The nuclear fraction was spun down once more for 5 min at 500*g*. Supernatant was removed from the nuclear fraction pellet and 500 μl of TRIzol (15596018, Invitrogen) was added to the nuclear fraction pellet. To both TRIzol homogenizations was added 1 μl of GlycoBlue coprecipitant (AM9516, Thermo Fisher Scientific). Then, RNA was extracted by phenol–chloroform extraction, followed by ethanol precipitation.

cDNA synthesis was carried out using the ProtoScript II first-strand cDNA synthesis kit (E6560L, New England Biolabs) with 6 μl of RNA in a 20-μl total volume using random hexamer primers supplied with the kit. Before qPCR, nuclear fraction cDNA was diluted 1:2 with nuclease-free water and cytosolic fraction cDNA was diluted 1:60 with nuclease-free water. qPCR with 500 nM of specified primers (Supplementary Table 3) was carried out using PowerTrack SYBR green master mix (A46109, Thermo Fisher Scientific) using the CFX Opus 384 (Bio-Rad) and qPCR parameters of 95 °C for 2 min, followed by 40 cycles of 95 °C for 15 s and 60 C°C for 1 min. From the qPCR *C*_*q*_ values, subcellular (nuclear and cytosolic), guide and *NEAT1* expression levels were normalized relative to their subcellular *GAPDH* expression controls; then, the ratio of these normalized subcellular expressions was calculated. Data were analyzed and plotted in Matlab (version R2024a).

### Splicing isoform quantification

For splicing isoform quantification, cDNA synthesis was carried out using the ProtoScript II first-strand cDNA synthesis kit (E6560L, New England Biolabs) with 3 μl of RNA in a 10-μl total volume using oligo(dT) primers supplied with the kit. PCR with 500 nM of specified primers (Supplementary Table 3) was carried out using NEBNext Ultra II Q5 master mix (M0544L, New England Biolabs) with a *T*_m_ of 68 °C and 30-s extension time for 32 cycles. PCR products were purified by a QIAquick PCR purification kit (28106, Qiagen), with 50% of eluted volume run on 2% E-Gel EX agarose gels(G402022, Thermo Fisher Scientific) for ~15 min with E-Gel ultralow-range DNA ladder (10488096, Thermo Fisher Scientific) and E-Gel 50-bp DNA ladder (10488099, Thermo Fisher Scientific). Gels were visualized using the Azure Biosystems c600. Gel bands were quantitated using GelAnalyzer (version 19.1), with PSI values calculated after adjusting bands for relative molecular weights.

### Luciferase reporter assay

The cell medium was changed 48 h after transfection. Luminescence was measured using a Tekan Infinite 200Pro plate reader (Costar 96 flat white setting, automatic attenuation) and Promega’s Dual-Glo luciferase assay system (E2920). Integration times were 500 ms for FLuc and 100 ms for RLuc. Data were analyzed and plotted in Matlab (version R2024a).

### In vitro transcription of pseudouridine standards

Template DNA was first generated by a two-stage PCR on a DNA oligo containing the respective target sequence context (Supplementary Table 3). First, the oligo was PCR-amplified for two cycles with pseudouridylation standard primers (PCR1). This PCR amplicon was then used as a template for a 15-cycle reaction with PCR2. The resulting PCR amplicon was PAGE-purified and divided into two T7 in vitro transcription reactions (MEGAshortscript, Invitrogen), containing the standard triphosphate pool (for unmodified standards) or with substitution of UTP for ΨTP (for pseudouridine standards). After a 120-min in vitro transcription, the reaction was treated with DNase (Turbo DNase, Invitrogen) and RNA was purified by PAGE before use in CMC or BID-seq reactions.

### Targeted BID-seq

BID-seq^33^ began with bisulfite treatment using 8.5 μl of DNaseI-treated total RNA, mixing it with 45 μl of 2.4 M Na_2_SO_3_ and 0.36 M NaHSO_3_ and heating at 70 °C for 3 h. The reaction was diluted with 75 μl of RNase-free water, combined with 270 μl of RNA-binding buffer and 400 μL ethanol and loaded onto a Zymo RNA clean and concentrator-5 column. The column was washed with 200 μl of RNA wash buffer and then incubated for 90 min at room temperature with 200 μl of RNA desulfonation buffer. After clearing the desulfonation buffer, the column was washed twice with 700 μl of RNA wash buffer. Bisulfite-treated RNA was eluted in 10.5 μl of nuclease-free water. For RT, 5 μl of treated RNA was combined with 1 μl of 50 mM random hexamers or 10 mM dNTPs and 6 μl of water, annealed at 65 °C for 5 min and snap-chilled. The primer-annealed RNA underwent RT with 4 μl of 5× SSIV buffer, 100 mM DTT, 1 μl of RNasein Plus and 1 μl of SSIV, incubated at 23 °C for 10 min, 55 °C for 10 min and 80 °C for 10 min. RNA hydrolysis involved adding 5 μl of 1 M NaOH and heating at 95 °C for 5 min, before quenching with 5 μl of 1 M HCl. The resulting cDNA was purified using MyOne Silane Dynabeads and eluted in 20 μl of nuclease-free water. Gene-specific PCR amplification used primers from Supplementary Table 3. PCR products were purified with AMPure XP beads, pooled equally and sent for amplicon sequencing. Raw FASTQ files were aligned to target references using bbmap; BAM files were sorted and indexed with SAMtools. Deletion rates were quantified per position using SAMtools mpileup and custom Python scripts. Effective pseudouridylation rates were calculated by normalizing to the 100% ΨTP *CFTR* synthetic RNA standard. Data were analyzed and plotted in Matlab (version R2024a).

### Targeted amplicon CMC sequencing

To disrupt RNA secondary structure, 5 μg of RNA in ~10 μl of nuclease-free water was incubated at 80 °C for 5 min and then chilled on ice. Denatured RNA was transferred to 100 μl of BEU buffer (50 mM bicine pH 8.5, 4 mM EDTA and 7 M urea) with 0.2 M CMC (C106402-1G, Sigma-Aldrich), incubated at 37 °C for 20 min and purified by ethanol precipitation. Pellets were dissolved in 50 μl of Na_2_CO_3_ buffer (50 mM Na_2_CO_3_ pH 10.4 and 2 mM EDTA), incubated at 37 °C for 2 h and purified by ethanol precipitation. Pellets were redissolved in 10 μl of nuclease-free water. For RT, 2 μl of random hexamer primers from the SuperScript first-strand synthesis system for RT–PCR (11904018, Thermo Fisher Scientific) were added, denatured at 65 °C for 5 min and chilled on ice. Then, 8 μl of freshly prepared 2.5× RT buffer (125 mM Tris pH 8.0, 15 mM MnCl_2_, 187.5 mM KCl, 1.25 mM dNTPs and 25 mM DTT) was added before incubating at 25 °C for 2 min. Next, 1 μl of SuperScript II reverse transcriptase from the SuperScript first-strand synthesis system for RT–PCR (11904018, Thermo Fisher Scientific) was added. Reactions ran at 25 °C for 10 min, 42 °C for 3 h and 70 °C for 15 min.

Library preparation involved two PCR steps. First, PCR with 500 nM locus-specific primers (Supplementary Table 3) used 1 μl of cDNA with NEBNext Ultra II Q5 master mix (M0544L, New England Biolabs) in 10 μl (*T*_m_ 65 °C) with a 30-s extension for 15 cycles. Products were cleaned with 1.8× AMPure XP beads (A63881, Beckman Coulter), eluted in 11 μl. Second, PCR with 500 nM next-generation sequencing universal barcoded primers (Supplementary Table 3) used 10 μl of first-round product with NEBNext Ultra II Q5 master mix (M0544L, New England Biolabs) in 20 μl (*T*_m_ 65 °C) with a 30-s extension for 15 cycles. Products were pooled, purified using the QIAquick PCR purification kit (28106, Qiagen) and run on 2% E-Gel EX agarose gels (G402022, Thermo Fisher Scientific) for ~15 min. The ~250-bp upper band was extracted and purified using the QIAquick gel extraction kit (28704, Qiagen) before sequencing. The library was sequenced on an Illumina NovaSeq X Plus 10B (150-bp paired-end reads), targeting ~5 million reads per sample.

FASTQ files were collapsed along UMI (unique molecular identifier) using the first occurrence of a 10-mer UMI. Only sequences with perfect 8-mer matches upstream or downstream of target NΨN were considered. Deletion rates were calculated as the fraction of total sequences at a locus with 2 nt between perfect 8-mer matches. Mutation rates were calculated as the fraction of total sequences at a locus with 3 nt between perfect 8-mer matches and no T at the Ψ position in target NΨN. Effective pseudouridylation rates were normalized to 100% ΨTP *ACTB*, *EEF2* and *RPS6* synthetic RNA standards. Data were analyzed and plotted in Matlab (version R2024a).

### Guide-specific qPCR to quantitate construct expression

First, 48 h after transfection, HEK293T cells were washed twice with PBS; then, RNA was extracted using the RNeasy Plus mini kit (74136, Qiagen) with an elution volume of 30 μl. cDNA synthesis was carried out using the ProtoScript II first-strand cDNA synthesis kit (E6560L, New England Biolabs) with 3 μl of RNA in a 10-μl total volume using two different reactions per replicate: random hexamer primers supplied with the kit for *GAPDH* quantitation and the primer used as the reverse qPCR primer for guide quantification (Supplementary Table 3). Before qPCR, cDNA was diluted 1:3 with nuclease-free water. qPCR with 500 nM specified primers (Supplementary Table 3) was carried out using the PowerUp SYBR green master mix (A25742, Thermo Fisher Scientific) with the CFX96 (Bio-Rad) and qPCR parameters of 50 °C for 2 min and 95 °C for 2 min, followed by 40 cycles of 95 °C for 15 s and 60 °C for 1 min. From the qPCR *C*_*q*_ values, guide expression levels were normalized relative to *GAPDH*. Data were analyzed and plotted in Matlab (version R2024a).

### RCA FISH experiment

U-2 OS cells (<20 passages) were seeded into 96-well glass-bottom microplates (Greiner Bio-One SensoPlate, 07-000-109). Plasmids were transfected into ~30% confluent cells using jetOPTIMUS DNA transfection reagent (76299-632, VWR International). Three bioreplicate wells per condition received 12.5 ng of plasmid DNA for A>I RNA RCA FISH and 25 ng for U>Ψ RNA RCA FISH. Cells were cultured to ~80% confluency and then incubated at 65 °C for 5 min, followed by rapid ice cooling before fixation. Cells were fixed for 30 min at room temperature in 4% (w/v) paraformaldehyde (PFA; Electron Microscopy Sciences, 15714) and 0.007% (v/v) glutaraldehyde (Electron Microscopy Sciences, 16120) in 1× PBS (Ambion, AM9625). After three 1× PBS washes, cells were permeabilized with 0.5% (v/v) Triton X-100 in 1× PBS for 10 min at room temperature. Permeabilization solution was removed by three washes with PBS containing 0.05% (v/v) Tween-20 (PBS-T; VWR, 100216-360).

Target snRNAs and snoRNAs were reverse-transcribed in situ. Each 50-μl well reaction contained 1× SuperScript IV buffer (Lifetech, 18090050), 500 μM of each dNTP (New England Biolabs, N0447L), 1 μM RT primer (Integrated DNA Technologies), 0.2 mg ml^−1^ BSA (New England Biolabs, B9200S), 0.8 U per μl RNase inhibitor (M0314L or Thermo Fisher Scientific, EO0384) and 20 U per μl of SuperScript IV reverse transcriptase (Lifetech, 18090050) in RNase-free water. Plates were sealed and incubated overnight (approximately 16–18 h) at 37 °C. Following RT, cells were washed three times with PBS-T and postfixed for 30 min at room temperature in 1× PBS with 3% (w/v) PFA and 0.1% (v/v) glutaraldehyde. Cells were washed five times with PBS-T. Padlock probe ligation was performed in a 50-μl well reaction with 1× Ampligase buffer (Lucigen, A3210K), 100 nM padlock probe (Integrated DNA Technologies, high-performance liquid chromatography (HPLC)-purified), 0.2 mg ml^−1^ BSA, 0.4 U per μl of RNase H (Enzymatics, Y9220L) and 0.5 U per μl of Ampligase (Lucigen, A3210K). The reaction incubated at 37 °C for 30 min and then 45 °C for 45 min on a thermocycler with a heated lid. Cells were then washed three times with PBS-T.

Ligated padlock probes were amplified by RCA. The 50-μl reaction mix per well, prepared on ice, contained 1× Phi29 buffer (Thermo Fisher Scientific, EP0091), 5% (v/v) glycerol (Sigma-Aldrich, G5516), 250 μM of each dNTP, 0.2 mg ml^−1^ BSA and 1 U per μl Phi29 DNA polymerase (Thermo Fisher Scientific, EP0091) (added last to chilled mixture). Plates were sealed (adjacent empty wells contained water for humidity) and incubated overnight (approximately 18 h) at 30 °C. After RCA, cells were washed three times with PBS-T. RCA products were detected by hybridization with fluorescently labeled hybridization probes (Integrated DNA Technologies, HPLC-purified). The 100-μl hybridization mix per well contained 1 μM hybridization probe in hybridization buffer (2× saline sodium citrate (Ambion, AM9763) and 10% (v/v) formamide). Hybridization was for 30 min at room temperature, followed by three PBS-T washes. Nucleoli were stained with 1 μM nucleolar red in 1× PBS for 5 min. Nuclei were counterstained with DAPI.

Fluorescence imaging used a Squid microscopy system (Cephalogics) with a pentaband filter set (laser lines at 405, 470, 550, 640 and 730 nm) and an IMX571 camera. An Olympus ×10 (0.8 numerical aperture) Plan Apo objective was used. Excitation wavelengths were 405 nm (DAPI), 561 nm (nucleolar red) and 638 nm (TYE665). For A>I RNA RCA FISH, nine images (~10,000–20,000 cells total) were taken per well. For U>Ψ RNA RCA FISH, 13 images (~20,000–40,000 cells total) were taken per well.

### RCA FISH analysis

RCA FISH images were analyzed using a custom CellProfiler (version 4.2.8) pipeline. Nuclei were identified in 405 filter images (10–40-pixel diameter) using IdentifyPrimaryObjects with a global, minimum cross-entropy thresholding method (smoothing scale, 1.3488; correction factor, 1.0; bounds, 0.2–1.0). Clumped objects were divided by shape and intensity. Cell boundaries were identified by expanding 20 pixels using ExpandOrShinkObjects. RCA rolonies were identified in 640 filter images (1–10-pixel diameter) using IdentifyPrimaryObjects with similar global, minimum cross-entropy thresholding (smoothing scale, 1.3488; correction factor, 1.0; bounds, 0.07–1.0). Clumped objects were divided by shape and intensity. Only rolonies within cell boundaries were counted (RelateObjects). For U>Ψ RNA RCA FISH images, nucleoli were identified in 550 filter images (1–7-pixel diameter) using IdentifyPrimaryObjects with an adaptive, robust background thresholding method (outlier fractions, 0.05; mean and s.d. methods, 1.5 deviations; smoothing scale, 0.674; correction factor, 1; bounds, 0.02–1.0; adaptive window, 7). Clumped objects were divided by intensity. Only nucleoli within nuclei were counted (RelateObjects).

Edge-to-edge distances between RCA rolonies and nuclei and nucleoli were determined first by binarizing nuclei and nucleoli (ConvertObjectsToImage) and then inverting (ImageMath, invert operation, standard scaling and clamping). A distance map was generated from these inverted images using Morph (distance operation). Finally, MeasureObjectIntensity calculated edge-to-edge distances as Intensity_MinIntensityEdge values, with 0 assigned if rolonies were within nuclei or nucleoli. RCA rolonies were considered localized within nuclei or nucleoli if they were children of nuclei or nucleoli (RelateObjects). Data were analyzed and plotted in Matlab (version R2024a).

### Cystic fibrosis disease modeling

Lentivirus was produced by seeding 8 × 10^6^ LentiX cells in a 10-cm dish 24 h before transfection. Virus particles were packaged using standard three-plasmid transfection with VirusGen reagent. Supernatants were collected 72 h after transfection, filtered (0.45-μm PVDF), concentrated by PEG precipitation (LentiX concentrator), snap-frozen and stored at −80 °C. Functional titers were determined on HEK293T cells by eGFP flow cytometry. For transduction, 200,000 16HBE14o^−^ *CFTR*^W1282X^ cells were seeded on ECM-coated 48-well plates 24 h beforehand. Cells were spin-transduced with titer-adjusted virus in PBS containing 5 μg ml^−1^ polybrene at 1,000 rcf for 15 min at 37 °C. The medium was changed after 48 h. Cultures were expanded over 3 weeks to 10-cm dishes, with 1.5 μg ml^−1^ puromycin selection on days 7–14 to prevent nontransduced cell expansion. Cells were then washed, scraped, pelleted and snap-frozen.

RNA was extracted from cell pellets using the RNeasy Plus mini kit (74136, Qiagen) with an elution volume of 30 μl. cDNA synthesis was carried out using the ProtoScript II first-strand cDNA synthesis kit (E6560L, New England Biolabs) with 6 μl of RNA in a 20-μl total volume using oligo(dT) primers supplied with the kit. qPCR with 500 nM of specified primers (Supplementary Table 3) was carried out using PowerTrack SYBR green master mix (A46109, Thermo Fisher Scientific) with the CFX Opus 384 (Bio-Rad) and qPCR parameters of 95 °C for 2 min, followed by 40 cycles of 95 °C for 15 s and 60 C°C for 1 min. From the qPCR *C*_*q*_ values, *CFTR* and *PuroR* expression levels were normalized relative to *GAPDH* and *ANXA5* housekeeping genes. Data were analyzed and plotted in Matlab (version R2024a).

### Reporting summary

Further information on research design is available in the Nature Portfolio Reporting Summary linked to this article.

## Online content

Any methods, additional references, Nature Portfolio reporting summaries, source data, extended data, supplementary information, acknowledgements, peer review information; details of author contributions and competing interests; and statements of data and code availability are available at 10.1038/s41589-025-02026-8.

## Supplementary information

Supplementary Information Supplementary Figs. 1–5, Dataset 1 (legend) and Tables 1–3.

Reporting Summary

Supplementary Dataset 1 DESeq2 output for Fig. 2. Spreadsheet containing standard DESeq2 output for conditions tested in Fig. 2 (RAB7A-targeting cadRNA, RAB7A-targeting U7smOPT snRNA, DMD-targeting cadRNA and DMD-targeting U7smOPT snRNA) relative to pUC19 control.

## Source data

Source Data Fig. 4 Uncropped scans of gels for Fig. 4.

## Data Availability

RNA-seq data from this study are available from the National Center for Biotechnology Information’s Gene Expression Omnibus under accession number GSE295421. Datasets from GENCODE Human Release 44 (GRCh38.p14) were used in this study. Uncropped scans of gels for Fig. 4 annotated with conditions and biological replicates are provided in Supplementary Figs. 4 and 5. Source data are provided with this paper.

## Code availability

Critical code used for data analysis can be accessed via Zenodo at 10.5281/zenodo.16755321 (ref. ^43^).
