# Supplementary material for: Enhancing RNA base editing on mammalian transcripts with small nuclear RNAs
Source: Nat Chem Biol. 2025 Sep 18;22(6):995–1003. doi: 10.1038/s41589-025-02026-8 (PMC13003931; doi:10.1038/s41589-025-02026-8)
Supplement: Supplementary file 1 — Supplementary Figs. 1–5, Dataset 1 (legend) and Tables 1–3. [file 41589_2025_2026_MOESM1_ESM.pdf]

nature chemical biology

Supplementary information https://doi.org/10.1038/s41589-025-02026-8

Enhancing RNA base editing on mammalian transcripts with small nuclear RNAs

In the format provided by the authors and unedited

TABLE OF CONTENTS

- **Supplementary Figure 1: Principal Component Analysis (PCA) plot of RNA-guided A>I base editor RNA sequencing data**
- **Supplementary Figure 2: Alignment of RNA-guided A>I base editor RNA sequencing reads to *DMD* gene**
- **Supplementary Figure 3: Differential splicing analysis of RNA-guided A>I base editor RNA sequencing data**
- **Supplementary Figure 4: Splicing gels for A>I editing-targeted pre-mRNA of *DENND4A*, *FBXL4*, and *PDE4DIP***
- **Supplementary Figure 5: Splicing gels for A>I editing-targeted pre-mRNA of *AHCY*, *CTNNA1*, and *HSF1***
- **Supplementary Dataset 1: DESeq2 output for Fig. 2**
- **Supplementary Table 1: Plasmid sequences**
- **Supplementary Table 2: Guide and snoRNA sequences**
- **Supplementary Table 3: Oligonucleotide sequences for PCR, qPCR, Sanger sequencing, NGS, pseudouridylation standardization, and RCA FISH**

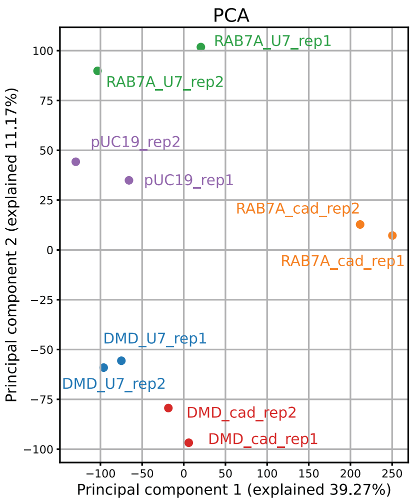

Supplementary Figure 1: Principal Component Analysis (PCA) plot of RNA-guided A>I base editor RNA sequencing data. PCA plot of RNA sequencing samples analyzed in Fig. 2, with each of two replicates colored by sample (pUC19, *RAB7A*-targeting cadRNA, *RAB7A*-targeting U7smOPT snRNA, *DMD*-targeting cadRNA, and *DMD*-targeting U7smOPT snRNA).

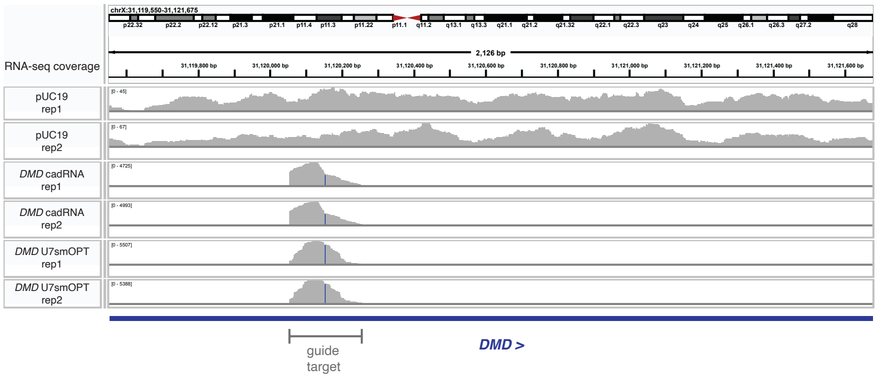

Supplementary Figure 2: Alignment of RNA-guided A>I base editor RNA sequencing reads to *DMD* gene. Alignment showing RNA sequencing read pile-up across *DMD*-targeting cadRNA and U7smOPT snRNA replicates at the guide target on *DMD*.

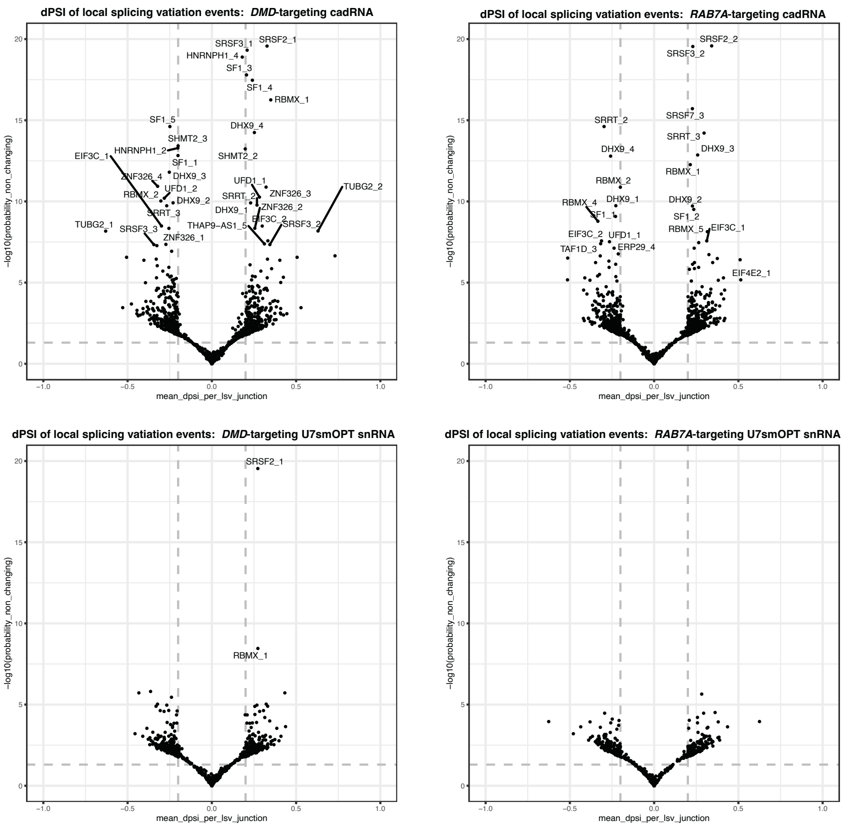

Supplementary Figure 3: Differential splicing analysis of RNA-guided A>I base editor RNA sequencing data. Scatterplots of local splicing variations (LSVs) against empty control (pUC19) of cadRNA backbone vs. U7smOPT snRNA backbone for *RAB7A*- and *DMD*-targeting guides. Cutoffs for significance are p-value < 0.05 and various dPSI (differential Percent Spliced In) values used in Fig. 2.

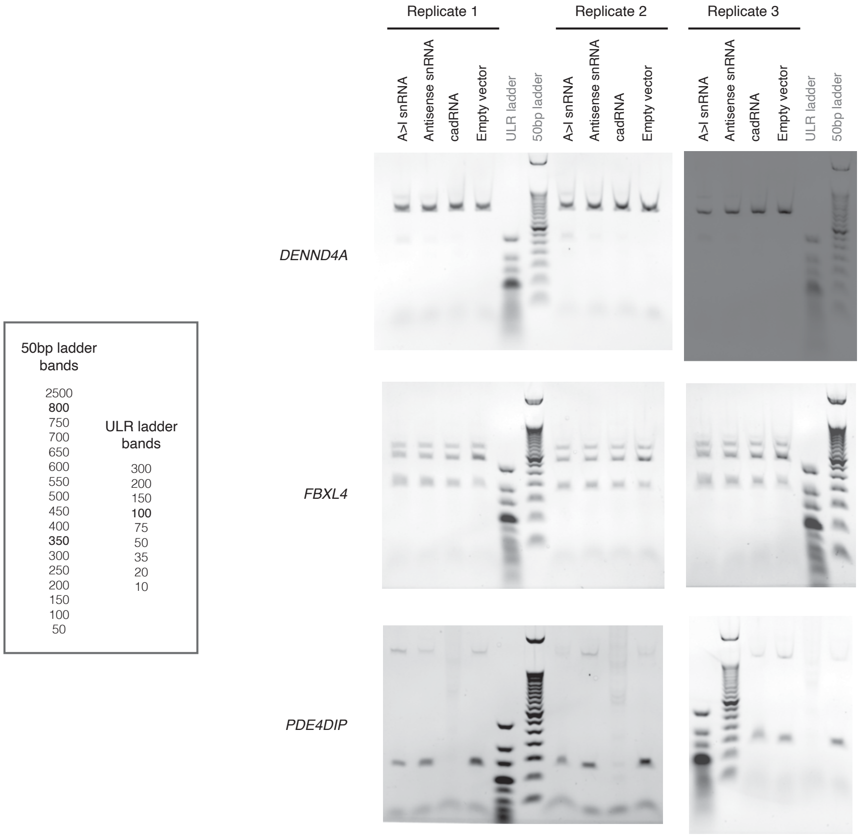

Supplementary Figure 4: Splicing gels for A>I editing-targeted pre-mRNA of *DENND4A*, *FBXL4*, and *PDE4DIP*. Full RT-PCR gels for all replicates of experiments in Fig. 4c. n = 3 biological replicates per condition.

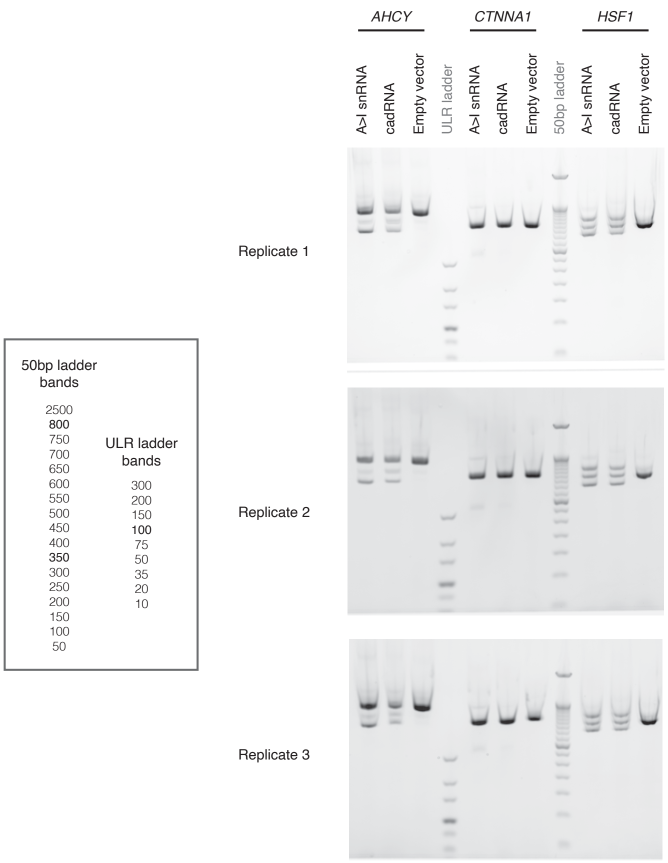

Supplementary Figure 5: Splicing gels for A>I editing-targeted pre-mRNA of *AHCY*, *CTNNA1*, and *HSF1*. Full RT-PCR gels for all replicates of experiments in Figure 4d. n = 3 biological replicates per condition.

Supplementary Dataset 1: DESeq2 output for Fig. 2.

Spreadsheet containing standard DESeq2 output for conditions tested in Fig. 2 (RAB7A-targeting cadRNA, RAB7A-targeting U7smOPT snRNA, DMD-targeting cadRNA, and DMD-targeting U7smOPT snRNA) relative to pUC19 control.

Supplementary Table 1: Plasmid sequences.

Name	Sequence
**U1 promoter-terminator-U1_snRNA backbone**	taacacaggctaaggaccagcttctttgggagagaacagacgcaggggcgggagggaaaagggagaggca<br>gacgtcacttccccttggcggctctggcagcagattggtcggttgagtggcagaaaggcagacggggactggg<br>caaggcactgtcggtgacatcacggacagggcgacttctatgtagatgaggcagcgcagaggctgctgcttcgc<br>cacttgctgcttcaccacgaaggagttcccgtgccctgggagcgggttcaggaccgctgatcggaagtgagaat<br>cccagctgtgtgtcagggctggaaagggctcgggagtgcgcggggcaagtgaccgtgtgtgtaaagagtgag<br>gcgtatgaggctgtgtcggggcagaggcccaagatctca**<guide>**gcaggggagataccatgatcacgaag<br>gtggttttcccagggcgaggcttatccattgcactccggatgtgctgacccctgcgatttccccaaatgtgggaaac<br>tcgactgcataatttgtggtagtgggggactgcgttcgcgctttcccctgactttctggagtttcaaaagtagactgta<br>cgctaa
**U7 promoter-terminator-U7smOPT_snRNA backbone**	ggcttaacaacaacgaaggggctgtgactggctgctttctcaaccaatcagcaccgaactcatttgcatgggctga<br>gaacaaatgttcgcgaactctagaaatgaatgacttaagtaagttccttagaatattatttttcctactgaaagttacca<br>catgcgtcgttgtttatacagtaataggaacaagaaaaaagtcacctaagctcaccctcatcaattgtggagttccttt<br>atatcccatcttctctccaaacacatacgcagc**<guide>**agaatttttggagtaggctttctggctttttaccggaa<br>agcccctcttatgatgtttgttgccaatgatagattgttttcactgtgcaaaaattatgggtagttttggtggtcttgatgc<br>agttgtaagcttggggtatgaaggtttgggccacgcctggg
**U1 promoter-terminator-U7smOPT_snRNA backbone**	taacacaggctaaggaccagcttctttgggagagaacagacgcaggggcgggagggaaaagggagaggca<br>gacgtcacttccccttggcggctctggcagcagattggtcggttgagtggcagaaaggcagacggggactggg<br>caaggcactgtcggtgacatcacggacagggcgacttctatgtagatgaggcagcgcagaggctgctgcttcgc<br>cacttgctgcttcaccacgaaggagttcccgtgccctgggagcgggttcaggaccgctgatcggaagtgagaat<br>cccagctgtgtgtcagggctggaaagggctcgggagtgcgcggggcaagtgaccgtgtgtgtaaagagtgag<br>gcgtatgaggctgtgtcggggcagaggcccaagatctca**<guide>**agaatttttggagtaggctttctggctttt<br>taccggaaagcccctactttctggagtttcaaaagtagactgtacgctaa
**U6 promoter-terminator-cadRNA backbone**	gagggcctatttcccatgattccttcatatttgcatatacgatacaaggctgttagagagataattagaattaatttgac<br>tgtaaacacaaagatattagtacaaaatacgtgacgtagaaagtaataatttcttgggtagtttgcagttttaaaattat<br>gttttaaaatggactatcatatgcttaccgtaacttgaaagtatttcgatttcttggctttatatatcttgtggaaaggacg<br>aaacaccgccatcagtcgccggtcccaagcccggataaaatgggagggggcgggaaaccgcctaaccatgcc<br>gactgatggcagaaaaaaaaaa**<guide>**aaaaaaaaaactgccatcagtcggcgtggactgtagaacactg<br>ccaatgccggtcccaagcccggataaaagtggagggtacagtccacgctttttt
**U6 promoter-terminator-for-**	gagggcctatttcccatgattccttcatatttgcatatacgatacaaggctgttagagagataattagaattaatttgac<br>tgtaaacacaaagatattagtacaaaatacgtgacgtagaaagtaataatttcttgggtagtttgcagttttaaaattat<br>gttttaaaatggactatcatatgcttaccgtaacttgaaagtatttcgatttcttggctttatatatcttgtggaaaggacg<br>**<snoRNA>**tttttt

**snoRNAs backbone**	
**U7 promoter-terminator-for-U>Ψ-snRNAs backbone**	ggcttaacaacaacgaaggggctgtgactggctgctttctcaaccaatcagcaccgaactcatttgcatgggctga<br>gaacaaatgttcgcgaactctagaaatgaatgacttaagtaagttccttagaatattatttttcctactgaaagttacca<br>catgcgtcgttgtttatacagtaataggaacaagaaaaaagtcacctaagctcaccctcatcaattgtggagttccttt<br>atatcccatcttctctccaaacacatacgcagc**<U>ΨsnRNA>**cttatgatgtttgttgccaatgatagattgtt<br>ttcactgtgcaaaaattatgggtagttttggtggtcttgatgcagttgtaagcttggggtatgaaggtttgggccacgc<br>ctggg
**CFTR PTC dual luciferase reporter**	GACATTGATTATTGACTAGTTATTAATAGTAATCAATTACGGGGTCATT<br>AGTTCATAGCCCATATATGGAGTTCCGCGTTACATAACTTACGGTAAAT<br>GGCCCGCCTGGCTGACCGCCCAACGACCCCCGCCCATTGACGTCAAT<br>AATGACGTATGTTCCCATAGTAACGCCAATAGGGACTTTCCATTGACG<br>TCAATGGGTGGAGTATTTACGGTAAACTGCCCACTTGGCAGTACATCA<br>AGTGTATCATATGCCAAGTACGCCCCCTATTGACGTCAATGACGGTAA<br>ATGGCCCGCCTGGCATTATGCCCAGTACATGACCTTATGGGACTTTCC<br>TACTTGGCAGTACATCTACGTATTAGTCATCGCTATTACCATGGTGATG<br>CGGTTTTGGCAGTACATCAATGGGCGTGGATAGCGGTTTGACTCACG<br>GGGATTTCCAAGTCTCCACCCCATTGACGTCAATGGGAGTTTGTTTTG<br>GCACCAAAATCAACGGGACTTTCCAAAATGTCGTAACAACTCCGCCC<br>CATTGACGCAAATGGGCGGTAGGCGTGTACGGTGGGAGGTCTATATA<br>AGCAGAGCTCTCTGGCTAACTAGAGAACCCACTGCTTACTGGCTTAT<br>CGAAATTAATACGACTCACTATAGGGAGACCCAAGCTGGCTAGCGTT<br>TAAACGGGCCCTCTAGACTCGAGCGGCCGCCACTGTGCTGGATATCT<br>GCAGGCCACCatggcttccaaggtgtacgaccccgagcaacgcaaacgcatgatcactgggcctcagt<br>ggtgggctcgctgcaagcaaatgaacgtgctggactccttcatcaactactatgattccgagaagcacgccgaga<br>acgccgtgatttttctgcatggtaacgctgcctccagctacctgtggaggcacgtcgtgcctcacatcgagcccgt<br>ggctagatgcatcatccctgatctgatcggaatgggtaagtccggcaagagcgggaatggctcatatcgcctcct<br>ggatcactacaagtacctcaccgcttggttcgagctgctgaaccttccaaagaaaatcatctttgtgggccacgact<br>gggggcttgtctggcctttcactactcctacgagcaccaagacaagatcaaggccatcgtccatgctgagagtg<br>tcgtggacgtgatcgagtcctgggacgagtggcctgacatcgaggaggatatcgccctgatcaagagcgaaga<br>gggcgagaaaatggtgcttgagaataacttcttcgtcgagaccatgctcccaagcaagatcatgcggaaactgga<br>gcctgaggagttcgctgcctacctggagccattcaaggagaagggcgaggttagacggcctaccctctcctggc<br>ctcgcgagatccctctcgttaagggaggcaagcccgacgtcgtccagattgtccgcaactacaacgcctaccttc<br>gggccagcgacgatctgcctaagatgttcatcgagtccgaccctgggttcttttccaacgctattgtcgagggagc<br>taagaagttccctaacaccgagttcgtgaaggtgaagggcctccacttcagccaggaggacgctccagatgaaa<br>tgggtaagtacatcaagagcttcgtggagcgcgtgctgaagaacgagcagaattctgcttgcaagaactggttca<br>gtagcttaagccactttgtgatccaccttaacagccacggcttccctcccgaggtggaggagcaggccgccggc<br>accctgcccatgagctgcgcccaggagagcggcatggatagacaccctgctgcttgcgccagcgccaggatca<br>acgtcAGCGGAAGTGAGACACCGGGTACGAGTGAGTCAGCTACTCCAG<br>AAAGT**gattcaataactttgcaacagtgAaggaaagcctttggagtgata**GGTTCTgccgatgct<br>aagaacattaagaagggccctgctcccttctaccctctggaggatggcaccgctggcgagcagctgcacaaggc<br>catgaagaggtatgccctggtgcctggcaccattgccttcaccgatgcccacattgaggtggacatcacctatgcc<br>gagtacttcgagatgtctgtgcgcctggccgaggccatgaagaggtacggcctgaacaccaaccaccgcatcgt<br>ggtgtgctctgagaactctctgcagttcttcatgccagtgctgggcgccctgttcatcggagtggccgtggcccct<br>gctaacgacatttacaacgagcgcgagctgctgaacagcatgggcatttctcagcctaccgtggtgttcgtgtcta

	agaagggcctgcagaagatcctgaacgtgcagaagaagctgcctatcatccagaagatcatcatcatggactcta<br>agaccgactaccagggcttccagagcatgtacacattcgtgacatctcatctgcctcctggcttcaacgagtacga<br>cttcgtgccagagtctttcgacagggacaaaaccattgccctgatcatgaacagctctgggtctaccggcctgcct<br>aagggcgtggccctgcctcatcgcaccgcctgtgtgcgcttctctcacgcccgcgaccctattttcggcaaccag<br>atcatccccgacaccgctattctgagcgtggtgccattccaccacggcttcggcatgttcaccaccctgggctacc<br>tgatttgcggctttcgggtggtgctgatgtaccgcttcgaggaggagctgttcctgcgcagcctgcaagactacaa<br>aattcagtctgccctgctggtgccaaccctgttcagcttcttcgctaagagcaccctgatcgacaagtacgacctgt<br>ctaacctgcacgagattgcctctggcggcgccccactgtctaaggaggtgggcgaagccgtggccaagcgcttt<br>catctgccaggcatccgccagggctacggcctgaccgagacaaccagcgccattctgattaccccagagggcg<br>acgacaagcctggcgccgtgggcaaggtggtgccattcttcgaggccaaggtggtggacctggacaccggca<br>agaccctgggagtgaaccagcgcggcgagctgtgtgtgcgcggccctatgattatgtccggctacgtgaataac<br>cctgaggccacaaacgccctgatcgacaaggacggctggctgcactctggcgacattgcctactgggacgagg<br>acgagcacttcttcatcgtggaccgcctgaagtctctgatcaagtacaagggctaccaggtggccccagccgag<br>ctggagtctatcctgctgcagcaccctaacattttcgacgccggagtggccggcctgcccgacgacgatgccgg<br>cgagctgcctgccgccgtcgtcgtgctggaacacggcaagaccatgaccgagaaggagatcgtggactatgtg<br>gccagccaggtgacaaccgccaagaagctgcgcggcggagtggtgttcgtggacgaggtgcccaagggcct<br>gaccggcaagctggacgcccgcaagatccgcgagatcctgatcaaggctaagaaaggcggcaagatcgccgt<br>gtaaGATCCGAGCTCGGTACCAAGCTTAAGTTTAAACCGCTGATCAGCC<br>TCGACTGTGCCTTCTAGTTGCCAGCCATCTGTTGTTTGCCCCTCCCCC<br>GTGCCTTCCTTGACCCTGGAAGGTGCCACTCCCACTGTCCTTTCCTAA<br>TAAAATGAGGAAATTGCATCGCATTGTCTGAGTAGGTGTCATTCTATT<br>CTGGGGGGTGGGGTGGGGCAGGACAGCAAGGGGGAGGATTGGGAA<br>GACAATAGCAGGCATGCTGGGGATGCGGTGGGCTCTATGG
**Lentiviral empty vector containing Ef1a_core-eGFP-PuroR cassette**	gaagatcctttgatcttttctacggggtctgacgctcagtggaacgaaaactcacgttaagggattttggtcatgaga<br>ttatcaaaaaggatcttcacctagatccttttaaattaaaaatgaagttttaaatcaatctaaagtatatatgagtaaactt<br>ggtctgacagttaccaatgcttaatcagtgaggcacctatctcagcgatctgtctatttcgttcatccatagttgcctg<br>actccccgtcgtgtagataactacgatacgggagggcttaccatctggccccagtgctgcaatgataccgcgaga<br>cccacgctcaccggctccagatttatcagcaataaaccagccagccggaagggccgagcgcagaagtggtcct<br>gcaactttatccgcctccatccagtctattaattgttgccgggaagctagagtaagtagttcgccagttaatagtttgc<br>gcaacgttgttgccattgctacaggcatcgtggtgtcacgctcgtcgtttggtatggcttcattcagctccggttccc<br>aacgatcaaggcgagttacatgatcccccatgttgtgcaaaaaagcggttagctccttcggtcctccgatcgttgtc<br>agaagtaagttggccgcagtgttatcactcatggttatggcagcactgcataattctcttactgtcatgccatccgtaa<br>gatgcttttctgtgactggtgagtactcaaccaagtcattctgagaatagtgtatgcggcgaccgagttgctcttgcc<br>cggcgtcaatacgggataataccgcgccacatagcagaactttaaaagtgctcatcattggaaaacgttcttcggg<br>gcgaaaactctcaaggatcttaccgctgttgagatccagttcgatgtaacccactcgtgcacccaactgatcttcag<br>catcttttactttcaccagcgtttctgggtgagcaaaaacaggaaggcaaaatgccgcaaaaaagggaataaggg<br>cgacacggaaatgttgaatactcatactcttcctttttcaatattattgaagcatttatcagggttattgtctcatgagcg<br>gatacatatttgaatgtatttagaaaaataaacaaataggggttccgcgcacatttccccgaaaagtgccacctgac<br>gtcgacggatcgggagatctcccgatcccctatggtgcactctcagtacaatctgctctgatgccgcatagttaag<br>ccagtatctgctccctgcttgtgtgttggaggtcgctgagtagtgcgcgagcaaaatttaagctacaacaaggcaa<br>ggcttgaccgacaattgcatgaagaatctgcttagggttaggcgttttgcgctgcttcgcgatgtacgggccagata<br>tacgcgttgacattgattattgactagttattaatagtaatcaattacggggtcattagttcatagcccatatatggagtt<br>ccgcgttacataacttacggtaaatggcccgcctggctgaccgcccaacgacccccgcccattgacgtcaataat<br>gacgtatgttcccatagtaacgccaatagggactttccattgacgtcaatgggtggagtatttacggtaaactgccc<br>acttggcagtacatcaagtgtatcatatgccaagtacgccccctattgacgtcaatgacggtaaatggcccgcctg<br>gcattatgcccagtacatgaccttatgggactttcctacttggcagtacatctacgtattagtcatcgctattaccatgg

	tgatgcggttttggcagtacatcaatgggcgtggatagcggtttgactcacggggatttccaagtctccaccccatt<br>gacgtcaatgggagtttgttttggcaccaaaatcaacgggactttccaaaatgtcgtaacaactccgccccattgac<br>gcaaatgggcggtaggcgtgtacggtgggaggtctatataagcagcgcgttttgcctgtactgggtctctctggtt<br>agaccagatctgagcctgggagctctctggctaactagggaacccactgcttaagcctcaataaagcttgccttga<br>gtgcttcaagtagtgtgtgcccgtctgttgtgtgactctggtaactagagatccctcagacccttttagtcagtgtgga<br>aaatctctagcagtggcgcccgaacagggacttgaaagcgaaagggaaaccagaggagctctctcgacgcag<br>gactcggcttgctgaagcgcgcacggcaagaggcgaggggcggcgactggtgagtacgccaaaaattttgact<br>agcggaggctagaaggagagagatgggtgcgagagcgtcagtattaagcgggggagaattagatcgcgatgg<br>gaaaaaattcggttaaggccagggggaaagaaaaaatataaattaaaacatatagtatgggcaagcagggagct<br>agaacgattcgcagttaatcctggcctgttagaaacatcagaaggctgtagacaaatactgggacagctacaacc<br>atcccttcagacaggatcagaagaacttagatcattatataatacagtagcaaccctctattgtgtgcatcaaaggat<br>agagataaaagacaccaaggaagctttagacaagatagaggaagagcaaaacaaaagtaagaccaccgcaca<br>gcaagcggccgctgatcttcagacctggaggaggagatatgagggacaattggagaagtgaattatataaatata<br>aagtagtaaaaattgaaccattaggagtagcacccaccaaggcaaagagaagagtggtgcagagagaaaaaag<br>agcagtgggaataggagctttgttccttgggttcttgggagcagcaggaagcactatgggcgcagcgtcaatgac<br>gctgacggtacaggccagacaattattgtctggtatagtgcagcagcagaacaatttgctgagggctattgaggc<br>gcaacagcatctgttgcaactcacagtctggggcatcaagcagctccaggcaagaatcctggctgtggaaagat<br>acctaaaggatcaacagctcctggggatttggggttgctctggaaaactcatttgcaccactgctgtgccttggaat<br>gctagttggagtaataaatctctggaacagatttggaatcacacgacctggatggagtgggacagagaaattaac<br>aattacacaagcttaatacactccttaattgaagaatcgcaaaaccagcaagaaaagaatgaacaagaattattgg<br>aattagataaatgggcaagtttgtggaattggtttaacataacaaattggctgtggtatataaaattattcataatgata<br>gtaggaggcttggtaggtttaagaatagtttttgctgtactttctatagtgaatagagttaggcagggatattcaccatt<br>atcgtttcagacccacctcccaaccccgaggggacccgacaggcccgaaggaatagaagaagaaggtggaga<br>gagagacagagacagatccattcgattagtgaacggatcggcactgcgtgcgccaattctgcagacaaatggca<br>gtattcatccacaattttaaaagaaaaggggggattggggggtacagtgcaggggaaagaatagtagacataata<br>gcaacagacatacaaactaaagaattacaaaaacaaattacaaaaattcaaaattttcgggtttattacagggacag<br>cagagatccagtttggttaattaaCGACGTTGTAAAACGACGGCCAGTGCCCTTTAT<br>GCGATTCAAGGTGCATATGGAAGGATCAGTCAATGGCCACGAATTTG<br>AGATCGAAGGTGTGGGGGAAGGAAAACCCTACGAAGGGACTCAGAC<br>TGCTAAGCTGCAGGTGACGAAAGGTGGCCCTCTTCCTTTCGCGTGGG<br>ACATTCTTTCCCCACAGTTCTTTTATGGGTCTAAGGCCTATATCAAACA<br>CCCCGCCGACATACCCGATTACCTCAAGCAAAGTTTTCCTGAAGGCT<br>TTAAGTGGGAGAGAGTCATGAATTTCGAGGATGGGGGCGTCGTGACC<br>GTGACCCAGGACTCCAGCCTGCAGGATGGCACCTTAATATATCATGTT<br>AAATTCATCGGAGTTAACTTCCCAAGCGATGGTCCGGTGATGCAAAA<br>GAAAACACTAGGTTGGGAGCCATCTACAGAGCGCAACTACCCACGT<br>GACGGCGTGCTCAAGGGGGAGAACCATATGGCATTGAAACTGAAGG<br>GAGGAGGGCACTACCTCTGCGAGTTCAAATCAATCTATATGGCTAAA<br>AAGCCGGTTAAGCTGCCTGGCTATCATTACGTAGACTACAAACTGGAT<br>ATTACCAGCCACAATGAAGATTACACGATCCTCTAGAGTCGACCTGC<br>AGGCATGCAAGCTTGGCGTAATCATGGTCATAGCTGTTTCCTCctagcatc<br>cgggcagagcAcacatcgcccacagtccccgagaagttggggggaggggtcggcaattgatccggtgccta<br>gagaaggtggcgcggggtaaactgggaaagtgatgtcgtgtactggctccgcctttttcccgagggtgggggag<br>aaccgtatataagtgcagtagtcgccgtgaacgttctttttcgcaacgggtttgccgccagaacacagactgcgat<br>cgcaatgtacagtatgaccgagtacaagcccacggtgcgcctcgccacccgcgacgacgtccccagggccgta<br>cgcaccctcgccgccgcgttcgccgactaccccgccacgcgccacaccgtcgatccggaccgccacatcgag

	cgggtcaccgagctgcaagaactcttcctcacgcgcgtcgggctcgacatcggcaaggtgtgggtcgcggacg<br>acggcgccgcggtggcggtctggaccacgccggagagcgtcgaagcgggggcggtgttcgccgagatcggc<br>ccgcgcatggccgagttgagcggttcccggctggccgcgcagcaacagatggaaggcctcctggcgccgcac<br>cggcccaaggagcccgcgtggttcctggccaccgtcggagtctcgcccgaccaccagggcaagggtctgggc<br>agcgccgtcgtgctccccggagtggaggcggccgagcgcgccggggtgcccgccttcctggagacctccgc<br>gccccgcaacctccccttctacgagcggctcggcttcaccgtcaccgccgacgtcgaggtgcccgaaggaccg<br>cgcacctggtgcatgacccgcaagcccggtgccggttccggcgcaacaaacttctctctgctgaaacaagccgg<br>agatgtcgaagagaatcctggaccggtgagcaagggcgaggagctgttcaccggggtggtgcccatcctggtc<br>gagctggacggcgacgtaaacggccacaagttcagcgtgtccggcgagggcgagggcgatgccacctacgg<br>caagctgaccctgaagttcatctgcaccaccggcaagctgcccgtgccctggcccaccctcgtgaccaccctga<br>cctacggcgtgcagtgcttcagccgctaccccgaccacatgaagcagcacgacttcttcaagtccgccatgccc<br>gaaggctacgtccaggagcgcaccatcttcttcaaggacgacggcaactacaagacccgcgccgaggtgaagt<br>tcgagggcgacaccctggtgaaccgcatcgagctgaagggcatcgacttcaaggaggacggcaacatcctgg<br>ggcacaagctggagtacaactacaacagccacaacgtctatatcatggccgacaagcagaagaacggcatcaa<br>ggtgaacttcaagatccgccacaacatcgaggacggcagcgtgcagctcgccgaccactaccagcagaacac<br>ccccatcggcgacggccccgtgctgctgcccgacaaccactacctgagcacccagtccgccctgagcaaagac<br>cccaacgagaagcgcgatcacatggtcctgctggagttcgtgaccgccgccgggatcactctcggcatggacg<br>agctgtacaagtaactcgagcgcgttaagtcgacaatcaacctctggattacaaaatttgtgaaagattgactggta<br>ttcttaactatgttgctccttttacgctatgtggatacgctgctttaatgcctttgtatcatgctattgcttcccgtatggctt<br>tcattttctcctccttgtataaatcctggttgctgtctctttatgaggagttgtggcccgttgtcaggcaacgtggcgtg<br>gtgtgcactgtgtttgctgacgcaacccccactggttggggcattgccaccacctgtcagctcctttccgggacttt<br>cgctttccccctccctattgccacggcggaactcatcgccgcctgccttgcccgctgctggacaggggctcggct<br>gttgggcactgacaattccgtggtgttgtcggggaaatcatcgtcctttccttggctgctcgcctgtgttgccacctg<br>gattctgcgcgggacgtccttctgctacgtcccttcggccctcaatccagcggaccttccttcccgcggcctgctg<br>ccggctctgcggcctcttccgcgtcttcgccttcgccctcagacgagtcggatctccctttgggccgcctccccgc<br>gtcgactttaagaccaatgacttacaaggcagctgtagatcttagccactttttaaaagaaaaggggggactggaa<br>gggctaattcactcccaacgaagacaagatctgctttttgcttgtactgggtctctctggttagaccagatctgagcc<br>tgggagctctctggctaactagggaacccactgcttaagcctcaataaagcttgccttgagtgcttcaagtagtgtg<br>tgcccgtctgttgtgtgactctggtaactagagatccctcagacccttttagtcagtgtggaaaatctctagcagggc<br>ccgtttaaacccgctgatcagcctcgactgtgccttctagttgccagccatctgttgtttgcccctcccccgtgccttc<br>cttgaccctggaaggtgccactcccactgtcctttcctaataaaatgaggaaattgcatcgcattgtctgagtaggtg<br>tcattctattctggggggtggggtggggcaggacagcaagggggaggattgggaagacaatagcaggcatgct<br>ggggatgcggtgggctctatggcttctgaggcggaaagaaccagctggggctctagggggtatccccacgcgc<br>cctgtagcggcgcattaagcgcggcgggtgtggtggttacgcgcagcgtgaccgctacacttgccagcgccta<br>gcgcccgctcctttcgctttcttcccttcctttctcgccacgttcgccggctttccccgtcaagctctaaatcgggggc<br>tccctttagggttccgatttagtgctttacggcacctcgaccccaaaaaacttgattagggtgatggttcacgtagtg<br>ggccatcgccctgatagacggtttttcgccctttgacgttggagtccacgttctttaatagtggactcttgttccaaact<br>ggaacaacactcaaccctatctcggtctattcttttgatttataagggattttgccgatttcggcctattggttaaaaaat<br>gagctgatttaacaaaaatttaacgcgaattaattctgtggaatgtgtgtcagttagggtgtggaaagtccccaggct<br>ccccagcaggcagaagtatgcaaagcatgcatctcaattagtcagcaaccaggtgtggaaagtccccaggctcc<br>ccagcaggcagaagtatgcaaagcatgcatctcaattagtcagcaaccatagtcccgcccctaactccgcccatc<br>ccgcccctaactccgcccagttccgcccattctccgccccatggctgactaattttttttatttatgcagaggccgag<br>gccgcctctgcctctgagctattccagaagtagtgaggaggcttttttggaggcctaggcttttgcaaaaagctccc<br>gggagcttgtatatccattttcggatctgatcagcacgtgttgacaattaatcatcggcatagtatatcggcatagtat<br>aatacgacaaggtgaggaactaaaccatggccaagttgaccagtgccgttccggtgctcaccgcgcgcgacgtc<br>gccggagcggtcgagttctggaccgaccggctcgggttctcccgggacttcgtggaggacgacttcgccggtgt

	ggtccgggacgacgtgaccctgttcatcagcgcggtccaggaccaggtggtgccggacaacaccctggcctgg<br>gtgtgggtgcgcggcctggacgagctgtacgccgagtggtcggaggtcgtgtccacgaacttccgggacgcct<br>ccgggccggccatgaccgagatcggcgagcagccgtgggggcgggagttcgccctgcgcgacccggccgg<br>caactgcgtgcacttcgtggccgaggagcaggactgacacgtgctacgagatttcgattccaccgccgccttctat<br>gaaaggttgggcttcggaatcgttttccgggacgccggctggatgatcctccagcgcggggatctcatgctggag<br>ttcttcgcccaccccaacttgtttattgcagcttataatggttacaaataaagcaatagcatcacaaatttcacaaataa<br>agcatttttttcactgcattctagttgtggtttgtccaaactcatcaatgtatcttatcatgtctgtataccgtcgacctcta<br>gctagagcttggcgtaatcatggtcatagctgtttcctgtgtgaaattgttatccgctcacaattccacacaacatacg<br>agccggaagcataaagtgtaaagcctggggtgcctaatgagtgagctaactcacattaattgcgttgcgctcactg<br>cccgctttccagtcgggaaacctgtcgtgccagctgcattaatgaatcggccaacgcgcggggagaggcggttt<br>gcgtattgggcgctcttccgcttcctcgctcactgactcgctgcgctcggtcgttcggctgcggcgagcggtatca<br>gctcactcaaaggcggtaatacggttatccacagaatcaggggataacgcaggaaagaacatgtgagcaaaag<br>gccagcaaaaggccaggaaccgtaaaaaggccgcgttgctggcgtttttccataggctccgcccccctgacgag<br>catcacaaaaatcgacgctcaagtcagaggtggcgaaacccgacaggactataaagataccaggcgtttccccc<br>tggaagctccctcgtgcgctctcctgttccgaccctgccgcttaccggatacctgtccgcctttctcccttcgggaa<br>gcgtggcgctttctcatagctcacgctgtaggtatctcagttcggtgtaggtcgttcgctccaagctgggctgtgtgc<br>acgaaccccccgttcagcccgaccgctgcgccttatccggtaactatcgtcttgagtccaacccggtaagacacg<br>acttatcgccactggcagcagccactggtaacaggattagcagagcgaggtatgtaggcggtgctacagagttct<br>tgaagtggtggcctaactacggctacactagaagaacagtatttggtatctgcgctctgctgaagccagttaccttc<br>ggaaaaagagttggtagctcttgatccggcaaacaaaccaccgctggtagcggtggtttttttgtttgcaagcagc<br>agattacgcgcagaaaaaaaggatctcaa

Supplementary Table 2: Guide and snoRNA sequences.

Name	Sequence
**RAB7A A>I guide**	AGACAGTTGTCCCCCTGGAGAGATGAAATCGATGTTGGCTCTTAATGGAAAGATAAAAGGCGTACATTCAAGATGTGTCTACTGTACAGAATACTGCCGCCAGCTGCTAATCCCAATTCTGAGTATGTGTCTGCAATCCAAACACCCATCAACCCTCCACCTTTGTCGCCTGCATTACAGGAGAATAACACATAATCCAA
**DAXX A>I guide**	GCTCCTGTAACCTGATGCCCACATCTCGGAAGGCATCCTGAGCCATGAGCTGGAGCTGCTGTCGGGGGAGGCCAAGGCTGTGTCGGGCAGCTGCCTTCTCCACAGCCCGAAGCACATCCCCATAGTCAGGGAAGGTATCAGGCCCTGGCTTGTTGATGAGCCGCTCAATGCGCCTGTTAACCTCTGGGTAGCGGGTGCCA
**GAPDH A>I guide**	GGCCATCCACAGTCTTCTGGGTGGCAGTCTACGCATGGACTGTGGTCTACTGTCCTTCCACGATACGTTCGTTGTCATGGATGACCTTGGCCAGGGGTGCCAAGCACAACGTGGTGCAGGAGGCAAACGTGATGATCTTGAGGCACAAGTCATACTTCTCATGCAAGACACCCATGACGAACATGGGGGCATCAGCAGAG
**TARDBP A>I guide**	TGTGTTTCATATTCCGTAAAACGAACAATCGGAAACCCCTTTGAATGTGGTGTCTTAAGATCTTTCAACTCCTGCACCATAAGAACTTCTCCAAAGGTACCAAAATTGAGTTTCAGGTCCTGTTCCCAAGTGTTCCATGGGAGAGGGTACACTATTAAATCGGTACAGTTCTGGACTGCTCTTGTCACTTTCACTGCTGA
**ALDOA A>I guide**	GTCCGTCCTTCTTGTACTGGGCACAGCGCTCAGACAGCCCATCCAACCCTTGGGTGGTAGTCTCGCCATTTGTCCCTGCCAGGGGGACCACGCCCTTGTCCACCTTGATGCCCACAACACCGCCCTTGGATTTGATAACTTGGGGGAAGGGACGCCCATCATCCGCCTTCTGGTAGAGTGTCTCATGGAAGAGGATGACAC
**SMAD4 A>I guide**	TTGTAGTCCACCATCCTGATAAGGTTAACCCGCCCAACGGTAAAAGAGGAGAGTCTAAAGGTTGTGCCAGTGCAATCGGCATGGTATGAAGTACTTCGTCCAGGAGGACCAGGGCCCGGTGTAAGACGGTTTCAATCCAGCAAGCACATTCTTTGATGCTCTGAGAAGGGTAATCCGGTCCCCAGCCTTTCACAAAACTC
**FANCC A>I guide**	GAGTCTGGGCTGAGGGACCTGGCTCTGCTAAATGTAAAATAGATACTTCGTGATTGTCCCAAGATGACATCAGCTCATTCTCACAGCCCAGCGAGGGCACCTACTCGTGTAATGCGTGGCCACAGCAGTTCACCTGTCCTGTGGGGGAGGCGAGCCTGATCCCAGTGGCCGGGCACCCACACGGCCTGCGTGCCTTCTAG
**GAA A>I guide**	tggtgtccagggggggccggcagcgtcacccactgcccctcgctgtggatggctggctcacggggagctgcaggtgggggtgggaggctgccaagggcctcCactggcaccgtctgcaggtcgtaccatgtgccccaaggggaagtagccagtcacttcggccttcccggcctggagcactggggtgatgagcagggcctccc
**BLM A>I guide**	gtggtgtaacaaatgattttgaagtctcagaagtatcaaagtcatccatatcatcccaatcattgatggtacttaaagaatctggtgaagaactaaattcCaatttcttgagagcagtatcccgggatttctttacagttggtgtgttttgggtagtgcatacaacttccttcggagtctgcaagaaatctggcaataatg

**CHD7 A>I guide**	gttcatactgattatagtgatcaaaatgtgtcagctttgtttgattttgattagttgaaggatgatgaagggat<br>ggctgtaaagaggcaaagccttggtcCattggcatttgctgacccataggatttactggattttccgggta<br>accacattctccgaggccttcaagaccttcactgaaaatattcccatcctcgccaa
**FANCD2 A>I guide**	acttaatctcttcaccctgcaagtcccggtttttagattgcccagccagaaagcctctctacaattgttgag<br>agtgagcatagctttgactctgcaaacCaaaagttccagggtctttttgagcagaggcacatgttgggtg<br>agtctcgtgtcctggtgaatcttggaatgcccacacaggtgatgaagcagccttgtgt
**COL4A5 A>I guide**	actgcatcctaggaattttatatatatatatatcacattttaaaacacaaaaggaattcttcaaaatgttatgtc<br>ctcttcatgcacacttgacatcggcCaattcgtgtcctcaagtctcctgctttcagcgtttctgactgaggtt<br>tactgaacatgtctgacacatctacagttgccagccaaaagctgtaggagttg
**DMD A>I guide**	aaagctaattacacttgatgtcagcccactctccaaaagctaattacacttgatgtcagaggtaacagattt<br>gcaaaattataggtcacacggtggtatcCattgaatgaatgatttaaaaatcaaaaagaaataaaatggc<br>atgaaagagtaaagctttttcctaccagtccttagcttttcctcttgagcttttctcct
**MDN1 A>I guide**	acaataaaatttttgtagttgtccaaaagggagcacctgggtaagcaatgtgaccttctgaccacagtta<br>agtctcactttggactcttctttctgttcCatgggtggtcagaggctgtcaccaactcaaaccactgtctga<br>gggcatcgctgagtgtctcaggaagtgcgtttacatctcgaagaatgatatagtatgg
**UBR4 A>I guide**	acaactcatggctcctaggtatgtacaggccctttgatggcttgggttacagacaacctcatagctggtgc<br>accacacacacgagataaaacaggaagccCaaaaaccccaagccacaccaagaaaaatgagagag<br>gggagggcggggtaacaatgcagcatcccgcggagggaacttaatgcacaaggagggagaacaga
**HOTAIR A>I guide**	ggaagttcaggcattgggaatggtaatctccatctgctgattttttttctgttcttctgtacttctattatttctgt<br>cttttaaataccccttctgtgtcCacatgcatcacttatttaagtgttctcctatgtctatttttctactgcaacttt<br>tgtcccaagctggggtctatatttagagtgcaaagtcccgtttgca
**MALAT1 A>I guide**	gaaaaatcttaaaaaaaggcttagcgcccacctcacccctccaccccccacgccaacacagtttgctcac<br>atgccagttactccagcataaagctgaaatcCattcaatactattgtcccataactgatctgactttgtatgt<br>aaatacagaaaaagcttgttcacctgttttcctcattttgtccactggtgaattcaact
**XIST A>I guide**	tgaataaacttgttaaatgacttttggtctggatctcacacctatattacttcatttccttctgtgagcactctat<br>aatgataacatcatttttatatccCagggcatgtagttccgagccccacagaaagtaatcaccattcagta<br>agccaatagttcattcctatctgtatagaactgtaggctttgtaaatctacaca
**DENND4A A>I guide**	gatgaggtactcccactaatatttgcggggcgcccatagggagtactctgaataatttcacaaccctgttt<br>caatctttctttccagtcatataaaacccCaaaaataaatttttaaaaataaatacacaaagcactttaaatat<br>gaaaagtaatattctcaggcatcatcaactgatccttaaatgtagtagcatggtaa
**FBXL4 A>I guide**	aggaagactggagactcctatccagcttcaaagcttcttgaaaatccaagcgaatgaagcaaatggaga<br>aggcaagggaaccaggccaggaagaaaaatcCaaaagcagagcaaaaatctcaaaaaccagaggt<br>aaacagtaccactctcttcgaggaatcaaggtagatctcaaaaatgcaaggaaataatccaaaaagt
**PDE4DIP A>I guide**	gagcctgaagtcttcgattgtcttccctgaggactctgttctcattgcagagctgaggtatggactccagg<br>ccctgactgtagaagttagaagtggatccCgtcccagaaagtagacatttgggtaacagaaactttcaa<br>aagtttcccattcgttatctctcaaagatactgccccaacccagcagaaaaccccaaggac
**DENND4A antisense guide**	gatgaggtactcccactaatatttgcggggcgcccatagggagtactctgaataatttcacaaccctgttt<br>caatctttctttccagtcatataaaaccctaaaaataaatttttaaaaataaatacacaaagcactttaaatat<br>gaaaagtaatattctcaggcatcatcaactgatccttaaatgtagtagcatggtaa
**FBXL4 antisense guide**	aggaagactggagactcctatccagcttcaaagcttcttgaaaatccaagcgaatgaagcaaatggaga<br>aggcaagggaaccaggccaggaagaaaaatctaaaagcagagcaaaaatctcaaaaaccagaggta<br>aacagtaccactctcttcgaggaatcaaggtagatctcaaaaatgcaaggaaataatccaaaaagt
**PDE4DIP antisense guide**	gagcctgaagtcttcgattgtcttccctgaggactctgttctcattgcagagctgaggtatggactccagg<br>ccctgactgtagaagttagaagtggatcctgtcccagaaagtagacatttgggtaacagaaactttcaaa<br>agtttcccattcgttatctctcaaagatactgccccaacccagcagaaaaccccaaggac

**AHCY A>I guide**	cacgaagctggggtggcccatggcacaacccaggttgaccagccgaccctcggccagcaggatgat<br>gcggcgcccattcttcaaccgataccggtccaccCacacgcaggcagggcaacagtgaaggcaggc<br>agggccccgctcccacagcccaccctctgatctggcaggcctctctgcctccaacagtgcccatgagg<br>a
**CTNNA1 A>I guide**	catcaaggcagccccactaatgatgctttccagacgctcctccagggaaggcctaaagcgctcctcgct<br>gaagctcaaggggtccacaatgatttgtttcCgcagagaaagtattaccgaatgttagtactcatctctgtt<br>cccttctttattcaaggtgagaaaagtgttattagtaatatcaagatttaacacaaaaat
**HSF1 A>I guide (~150 nt guide)**	tcgttccccctcacccccgcccgcctacgcacactggccaggctgctgtcaaggtcaggcaggctca<br>tgtcgggcacggtcaccgagggctgaacagcCgcggaggagggagggaagtcagaacagcacc<br>cgggggcggggcggggagg
**IDUA H/ACA box snoRNA**	GTGCACATcttcgccGACCTGCTTTCTTCTATGTGAGTAGTGTgactgc<br>ATGTGCTATACAAATAATTGAAGGctttggtcGCAGTATAACTATAAA<br>TAGTAATGCTGCgagttgcCCTTCAGACAAAA
**CFTR H/ACA box snoRNA**	GTGCACATCTTTCCTTGACCTGCTTTCTTCTATGTGAGTAGTGTC<br>CTGTTGCATGTGCTATACAAATAATTGAAGGCTTTCCTTGCAGT<br>ATAACTATAAATAGTAATGCTGCCTGTTCCCTTCAGACAAAA
**ACTB-U1226 H/ACA box snoRNA**	GTGCACATAACGCAACGACCTGCTTTCTTCTATGTGAGTAGTGT<br>CAGTCATAATGTGCTATACAAATAATTGAAGGACGCAACGCAGT<br>ATAACTATAAATAGTAATGCTGCAGTCAACCTTCAGACAAAA
**EEF2-U2881 H/ACA box snoRNA**	GTGCACATTAAGTCCCGACCTGCTTTCTTCTATGTGAGTAGTGT<br>CCTAAGAGATGTGCTATACAAATAATTGAAGGAAGTCCCGCAG<br>TATAACTATAAATAGTAATGCTGCCTAAGACCTTCAGACAAAA
**RPS6-U514 H/ACA box snoRNA**	GTGCACATGGCTTTCTGACCTGCTTTCTTCTATGTGAGTAGTGT<br>TCAACATAATGTGCTATACAAATAATTGAAGGCCTTTCTGCAGT<br>ATAACTATAAATAGTAATGCTGCCAACATCCTTCAGACAAAA

Supplementary Table 3: Oligonucleotide sequences for PCR, qPCR, Sanger sequencing, NGS, pseudouridylation standardization, and RCA FISH.

Name	Sequence
**RAB7A A>I FOR**	CCTCCCTCCTTGAAGGCTACC
**RAB7A A>I REV**	AAGCTCCGCTAACCTAAGAATACC
**GAPDH A>I FOR**	GATGCTGGCGCTGAGTACGT
**GAPDH A>I REV**	CACCACTGACACGTTGGCAGT
**TARDBP A>I FOR**	CCATCGGAAGACGATGGGACG
**TARDBP A>I REV**	CCTGAATGGCTTGGGGATGAAG
**FANCC A>I FOR**	CCTGCACAACAGCTGATCAGGC
**FANCC A>I REV**	TTCTTTAATGGTTCATGACCAAATTCTTGG
**ALDOA A>I FOR**	GACAGCTGACGACCGCGTGAACC
**ALDOA A>I REV**	CCCCAATCTTCAGCACACAACGCCA
**DAXX A>I FOR**	CTGACCGGCCGTGTCATAGAGCAGC
**DAXX A>I REV**	GTGAGGTGGCAGCCAAAGTTGTAGATGA
**SMAD4 A>I FOR**	GCGTGCACCTGGAGATGCTG
**SMAD4 A>I REV**	ACAGGTGAAGAATTAATAAGAATGTGTTTCTCCT
**GAA A>I FOR**	ttctaccccttcatgcggaac
**GAA A>I REV**	ggtgaagttggagacaggga
**BLM A>I FOR**	cccaacaccacaaatcagcaa
**BLM A>I REV**	aggagggtggaggcaaatca
**CHD7 A>I FOR**	gtgaagtgaagcacaggcaag
**CHD7 A>I REV**	gagcagtagaggggtggtga
**FANCD2 A>I FOR**	tcttccgtgtgatgatggct
**FANCD2 A>I REV**	tctcatctgctgtgctctcc
**COL4A5 A>I FOR**	agtttcgttcagctcccttca
**COL4A5 A>I REV**	ccgggatacagcaggattag
**MDN1 A>I FOR**	cagaggaagaccaagacccc
**MDN1 A>I REV**	AAAACCTCAGCCCAGGCAAA
**UBR4 A>I FOR**	GAGTACATCCGCCACAACGA
**UBR4 A>I REV**	agtatgaagctggccggga
**DMD A>I FOR**	GCGAGTAGTTCCACACAGGT
**DMD A>I REV**	TCAGGAACACCCCAAAACCA
**NEAT1 qPCR FOR**	GCTGGACCTTTCATGTAACGGG
**NEAT1 qPCR REV**	TGAACTCTGCCGGTACAGGGAA
**GAPDH qPCR FOR**	GTCTCCTCTGACTTCAACAGCG
**GAPDH qPCR REV**	ACCACCCTGTTGCTGTAGCCAA
**HOTAIR A>I FOR**	gcaccgcttttctaactggc
**HOTAIR A>I REV**	acacaaagtgcatacctaccca
**MALAT1 A>I FOR**	tgtgtgggtttctctctccc
**MALAT1 A>I REV**	ccgcttgagatttgggcttt
**XIST A>I FOR**	acgcaagggattggttgtct
**XIST A>I REV**	ctcaatcaggccaggaagca

**RAB7A guide qPCR FOR**	AGTTGTCCCCCTGGAGAGATG
**RAB7A guide qPCR REV**	CCTGTAATGCAGGCGACAAA
**GAPDH guide qPCR FOR**	CGCATGGACTGTGGTCTACT
**GAPDH guide qPCR REV**	CCATGTTCGTCATGGGTGTC
**TARDBP guide qPCR FOR**	GAGTTTCAGGTCCTGTTCCCA
**TARDBP guide qPCR REV**	AAGTGACAAGAGCAGTCCAGAA
**DENND4A A>I FOR**	GCCAAAATGCCCAGTTGCTT
**DENND4A A>I REV**	TACTCATTGCTTTTGTAGTGGGC
**FBXL4 A>I FOR**	TTCCTTCAGTTCCTTTTTCTGCTT
**FBXL4 A>I REV**	AGTGTCAGCATGGAAACTACTCA
**PDE4DIP A>I FOR**	GTAAGGGTCCTCCCACCTCT
**PDE4DIP A>I REV**	GCTGTGAGTTTGGGTTGGTG
**DENND4A RT-PCR FOR**	TTACCCCAACTGGATTGTCAG
**DENND4A RT-PCR REV**	TACAGTGTTCGTCTTTGCCAC
**FBXL4 RT-PCR FOR**	TTGTGCCGCATCTAGAGAGTC
**FBXL4 RT-PCR REV**	CCAGGTACCCTGAACTTTGC
**PDE4DIP RT-PCR FOR**	GAACACCGGCTGACCTCTAC
**PDE4DIP RT-PCR REV**	CTTCGATTGTCTTCCCTGAGGAC
**AHCY A>I FOR**	GAGACGGGCTTTCACTGTGTTG
**AHCY A>I REV**	AACGGGGTACTTGTCTGGATGG
**CTNNA1 A>I FOR**	GTAGGCCATCTTCTGTGGGACA
**CTNNA1 A>I REV**	TGTACTCCGAAAGCAGGTCCTG
**HSF1 A>I FOR**	CTGTTCTGACTTCCCTCCCTCC
**HSF1 A>I REV**	TGGGACTTGGCTCACCTGAATC
**AHCY RT-PCR FOR**	gtcaagtggctcaacgagaacg
**AHCY RT-PCR REV**	tccaagaccactgagctcatgg
**CTNNA1 RT-PCR FOR**	caccctgatgtcgcagcctata
**CTNNA1 RT-PCR REV**	ctgaaacgtggtccatgacagc
**HSF1 RT-PCR FOR**	tgcctggacaagaatgagctca
**HSF1 RT-PCR REV**	ctctaggagacagtggggtcct
**CFTR snoRNA guide FOR**	ACATCTTTCCTTGACCTGCTTTC
**CFTR snoRNA guide REV and cDNA primer**	AGGGAACAGGCAGCATTACTA
**ACTB snoRNA guide FOR**	CGCAACGACCTGCTTTCTTC
**ACTB snoRNA guide REV and cDNA primer**	ATACTGCGTTGCGTCCTTCA
**EEF2 snoRNA guide FOR**	TAAGTCCCGACCTGCTTTCTT
**EEF2 snoRNA guide REV and cDNA primer**	CTGAAGGTCTTAGGCAGCATT
**RPS6 snoRNA guide FOR**	CATGGCTTTCTGACCTGCTTT
**RPS6 snoRNA guide REV and cDNA primer**	TCTGAAGGATGTTGGCAGC
**PuroR qPCR FOR**	atgaccgagtacaagcccac
**PuroR qPCR REV**	acaccttgccgatgtcgag

**ANXA5 qPCR FOR**	CAAGCCTTTCATAGCCTTCC
**ANXA5 qPCR REV**	AGTCTGGTCCTGCTTCACCT
**Targeted amplicon CMC sequencing ACTB-U1226-TAG-F**	ACACTCTTTCCCTACACGACGCTCTTCCGATCTNNNNtccatcgtccaccgcaaatgct
**Targeted amplicon CMC sequencing ACTB-U1226-TAG-R**	TGGAGTTCAGACGTGTGCTCTTCCGATCTNNNNNNgccaatctcatcttgttttctgcgca
**Targeted amplicon CMC sequencing EEF2-U2881-TAG-F**	ACACTCTTTCCCTACACGACGCTCTTCCGATCTNNNNcagagtccggaggcagcag
**Targeted amplicon CMC sequencing EEF2-U2881-TAG-R**	TGGAGTTCAGACGTGTGCTCTTCCGATCTNNNNNNaaagtgttgggtgtcccatccc
**Targeted amplicon CMC sequencing RPS6-U514-TAA-F**	ACACTCTTTCCCTACACGACGCTCTTCCGATCTNNNNcgcaaacttttcaatctctcTAAa
**Targeted amplicon CMC sequencing RPS6-U514-TAA-R**	TGGAGTTCAGACGTGTGCTCTTCCGATCTNNNNNNaggacacgtggagtaacaag
**Targeted amplicon CMC sequencing Universal NGS Primer 1**	AATGATACGGCGACCACCGAGATCTACACTCTTTCCCTACACGACGCTCTTCCGATCT
**Targeted amplicon CMC sequencing Index NGS Primer 2-1**	CAAGCAGAAGACGGCATACGAGATCATACCACGTGACTGGAGTTCAGACGTGTGCTCTTCCGATC
**Targeted amplicon CMC sequencing Index NGS Primer 2-2**	CAAGCAGAAGACGGCATACGAGATGAAGTTGGGTGACTGGAGTTCAGACGTGTGCTCTTCCGATC
**Targeted amplicon CMC sequencing Index NGS Primer 2-3**	CAAGCAGAAGACGGCATACGAGATATGACGTCGTGACTGGAGTTCAGACGTGTGCTCTTCCGATC
**Targeted amplicon CMC sequencing Index NGS Primer 2-4**	CAAGCAGAAGACGGCATACGAGATTTGGACGTGTGACTGGAGTTCAGACGTGTGCTCTTCCGATC
**Targeted amplicon CMC sequencing Index NGS Primer 2-5**	CAAGCAGAAGACGGCATACGAGATAGTGGATCGTGACTGGAGTTCAGACGTGTGCTCTTCCGATC
**Targeted amplicon CMC sequencing Index NGS Primer 2-6**	CAAGCAGAAGACGGCATACGAGATGATAGGCTGTGACTGGAGTTCAGACGTGTGCTCTTCCGATC
**Targeted amplicon CMC sequencing Index NGS Primer 2-7**	CAAGCAGAAGACGGCATACGAGATTGGTAGCTGTGACTGGAGTTCAGACGTGTGCTCTTCCGATC
**Targeted amplicon CMC sequencing Index NGS Primer 2-8**	CAAGCAGAAGACGGCATACGAGATCGCAATCTGTGACTGGAGTTCAGACGTGTGCTCTTCCGATC
**Targeted amplicon CMC sequencing Index NGS Primer 2-9**	CAAGCAGAAGACGGCATACGAGATGATGTGTGGTGACTGGAGTTCAGACGTGTGCTCTTCCGATC

**Targeted amplicon CMC sequencing Index NGS Primer 2-10**	CAAGCAGAAGACGGCATACGAGATGATTGCTCGTGACTGGAGTTCAGACGTGTGCTCTTCCGATC
**Targeted amplicon CMC sequencing Index NGS Primer 2-11**	CAAGCAGAAGACGGCATACGAGATCGCTCTATGTGACTGGAGTTCAGACGTGTGCTCTTCCGATC
**Targeted amplicon CMC sequencing Index NGS Primer 2-12**	CAAGCAGAAGACGGCATACGAGATTATCGGTCGTGACTGGAGTTCAGACGTGTGCTCTTCCGATC
**Targeted amplicon CMC sequencing Index NGS Primer 2-13**	CAAGCAGAAGACGGCATACGAGATAACGTCTGGTGACTGGAGTTCAGACGTGTGCTCTTCCGATC
**Targeted amplicon CMC sequencing Index NGS Primer 2-14**	CAAGCAGAAGACGGCATACGAGATACGTTCAGGTGACTGGAGTTCAGACGTGTGCTCTTCCGATC
**Targeted amplicon CMC sequencing Index NGS Primer 2-15**	CAAGCAGAAGACGGCATACGAGATCAGTCCAAGTGACTGGAGTTCAGACGTGTGCTCTTCCGATC
**Targeted amplicon CMC sequencing Index NGS Primer 2-16**	CAAGCAGAAGACGGCATACGAGATTTGCAGACGTGACTGGAGTTCAGACGTGTGCTCTTCCGATC
**Targeted amplicon CMC sequencing Index NGS Primer 2-17**	CAAGCAGAAGACGGCATACGAGATCAATGTGGGTGACTGGAGTTCAGACGTGTGCTCTTCCGATC
**Targeted amplicon CMC sequencing Index NGS Primer 2-18**	CAAGCAGAAGACGGCATACGAGATACTCCATCGTGACTGGAGTTCAGACGTGTGCTCTTCCGATC
**Targeted amplicon CMC sequencing Index NGS Primer 2-19**	CAAGCAGAAGACGGCATACGAGATGTTGACCTGTGACTGGAGTTCAGACGTGTGCTCTTCCGATC
**Targeted amplicon CMC sequencing Index NGS Primer 2-20**	CAAGCAGAAGACGGCATACGAGATCGTGTGTAGTGACTGGAGTTCAGACGTGTGCTCTTCCGATC
**Targeted amplicon CMC sequencing Index NGS Primer 2-21**	CAAGCAGAAGACGGCATACGAGATACGACTTGGTGACTGGAGTTCAGACGTGTGCTCTTCCGATC
**Targeted amplicon CMC sequencing Index NGS Primer 2-22**	CAAGCAGAAGACGGCATACGAGATCACTAGCTGTGACTGGAGTTCAGACGTGTGCTCTTCCGATC
**Targeted amplicon CMC sequencing Index NGS Primer 2-23**	CAAGCAGAAGACGGCATACGAGATACTAGGAGGTGACTGGAGTTCAGACGTGTGCTCTTCCGATC
**Targeted amplicon CMC sequencing Index NGS Primer 2-24**	CAAGCAGAAGACGGCATACGAGATGTAGGAGTGTGACTGGAGTTCAGACGTGTGCTCTTCCGATC

**Targeted amplicon CMC sequencing Index NGS Primer 2-25**	CAAGCAGAAGACGGCATACGAGATCCTGATTGGTGACTGGAGTTCAGACGTGTGCTCTTCCGATC
**Targeted amplicon CMC sequencing Index NGS Primer 2-26**	CAAGCAGAAGACGGCATACGAGATATGCACGAGTGACTGGAGTTCAGACGTGTGCTCTTCCGATC
**Targeted amplicon CMC sequencing Index NGS Primer 2-27**	CAAGCAGAAGACGGCATACGAGATCGACGTTAGTGACTGGAGTTCAGACGTGTGCTCTTCCGATC
**Targeted amplicon CMC sequencing Index NGS Primer 2-28**	CAAGCAGAAGACGGCATACGAGATTACGCCTTGTGACTGGAGTTCAGACGTGTGCTCTTCCGATC
**Targeted amplicon CMC sequencing Index NGS Primer 2-29**	CAAGCAGAAGACGGCATACGAGATCCGTAAGAGTGACTGGAGTTCAGACGTGTGCTCTTCCGATC
**Targeted amplicon CMC sequencing Index NGS Primer 2-30**	CAAGCAGAAGACGGCATACGAGATATCACACGGTGACTGGAGTTCAGACGTGTGCTCTTCCGATC
**Targeted amplicon CMC sequencing Index NGS Primer 2-31**	CAAGCAGAAGACGGCATACGAGATCACCTGTTGTGACTGGAGTTCAGACGTGTGCTCTTCCGATC
**Targeted amplicon CMC sequencing Index NGS Primer 2-32**	CAAGCAGAAGACGGCATACGAGATCTTCGACTGTGACTGGAGTTCAGACGTGTGCTCTTCCGATC
**Targeted amplicon CMC sequencing Index NGS Primer 2-33**	CAAGCAGAAGACGGCATACGAGATTGCTTCCAGTGACTGGAGTTCAGACGTGTGCTCTTCCGATC
**Targeted amplicon CMC sequencing Index NGS Primer 2-34**	CAAGCAGAAGACGGCATACGAGATAGAACGAGGTGACTGGAGTTCAGACGTGTGCTCTTCCGATC
**Targeted amplicon CMC sequencing Index NGS Primer 2-35**	CAAGCAGAAGACGGCATACGAGATGTTCTCGTGTGACTGGAGTTCAGACGTGTGCTCTTCCGATC
**Targeted amplicon CMC sequencing Index NGS Primer 2-36**	CAAGCAGAAGACGGCATACGAGATTCAGGCTTGTGACTGGAGTTCAGACGTGTGCTCTTCCGATC
**Pseudouridylation standardization EEF2 standard (Ψ site underlined)**	CACAAAGAGCCGGACCACAGTAGAGAcGcAGGAcAcGAAGGccacacgggcaccaaggac
**Pseudouridylation standardization RPS6 standard (Ψ site underlined)**	CACAAAGAGCCGGACCACCGTAAAGAcGcAGGAcAcGAAGGccacacgggcaccaaggac
**Pseudouridylation standardization ACTB standard (Ψ site underlined)**	CACAAAGAGCCGGACCACCTTAGAGAcGcAGGAcAcGAAGGccacacgggcaccaaggac

**Pseudouridylation standardization CFTR standard (Ψ site underlined)**	CACAAAGAGCCGGACCACCGTGAAGAcGcAGGAcAcGAAGGccacacgggcaccaaggac
**Pseudouridylation standardization FOR primer for generating IVT template (PCR1)**	TAATACGACTCACTATAGGGAGGGAACAGAAACAGAGACTAGGAGGGGCGCGTAGGATAAATAATTCACACAAAGAGCCGGACCACA
**Pseudouridylation standardization FOR primer for generating IVT template (PCR2)**	TAATACGACTCACTATAGGGAGGGAACAGA
**Pseudouridylation standardization REV primer for generating IVT template (PCR1, PCR2)**	TGTGTGGTCCTTGGTGCCCGTGTG
**BID-Seq FOR primer for Ψ standard**	ACACTCTTTCCCTACACGACGCTCTTCCGATCTATAAACGGGCGGAGGGAACAGAAACAGAGA
**BID-Seq REV primer for Ψ standard**	GACTGGAGTTCAGACGTGTGCTCTTCCGATCTTATTAGTTATGTGTGGTCCTTGGTGCCCGTGTG
**BID-Seq FOR primer for CFTR luciferase reporter**	ACACTCTTTCCCTACACGACGCTCTTCCGATCTAAGTGAGACACCGGGTACGA
**BID-Seq REV primer for CFTR luciferase reporter**	GACTGGAGTTCAGACGTGTGCTCTTCCGATCTGCATCGGCAGAACCTATCACT
**RCA FISH GAPDH A>I RT primer**	A+GA+AG+TA+TG+AC+TT+GTGCCTCAAGAT
**RCA FISH GAPDH A>I padlock probe**	/5phos/AGGAGGCAAACGTGATGATCTTGAGctcctgttcgacacctacccacctcatcccactcttcaGTGCCAAGCACAACGTGGTGC
**RCA FISH GAPDH A>I hybridization probe**	/5TYE665/cacctcatcccactcttcaAGGAGGCAAACGTGA
**RCA FISH EEF2 U>Ψ RT primer**	A+CT+AT+TT+AT+AG+TT+ATACTGCGGGACTTC
**RCA FISH EEF2 U>Ψ padlock probe**	/5phos/AATAATTGAAGGAAGTCCCGCctcctgttcgacacctacccacctcatcccactcttcaGTCCTAAGAGATGTGCTATACA
**RCA FISH EEF2 U>Ψ hybridization probe**	/5TYE665/cacctcatcccactcttcaGTCCTAAGAGATGTG
