# Supplementary material for: Enhancing RNA base editing on mammalian transcripts with small nuclear RNAs
Source: Nat Chem Biol. 2025 Sep 18;22(6):995–1003. doi: 10.1038/s41589-025-02026-8 (PMC13003931; doi:10.1038/s41589-025-02026-8)
Supplement: Supplementary file 2 — Reporting Summary [file 41589_2025_2026_MOESM2_ESM.pdf]

Corresponding author(s): Gene W. Yeo

Last updated by author(s): Aug 6, 2025

Reporting Summary

Nature Portfolio wishes to improve the reproducibility of the work that we publish. This form provides structure for consistency and transparency in reporting. For further information on Nature Portfolio policies, see our Editorial Policies and the Editorial Policy Checklist.

Statistics

For all statistical analyses, confirm that the following items are present in the figure legend, table legend, main text, or Methods section.

n/a	Confirmed	
☐	☒	The exact sample size (*n*) for each experimental group/condition, given as a discrete number and unit of measurement
☐	☒	A statement on whether measurements were taken from distinct samples or whether the same sample was measured repeatedly
☐	☒	The statistical test(s) used AND whether they are one- or two-sided<br>*Only common tests should be described solely by name; describe more complex techniques in the Methods section.*
☒	☐	A description of all covariates tested
☐	☒	A description of any assumptions or corrections, such as tests of normality and adjustment for multiple comparisons
☐	☒	A full description of the statistical parameters including central tendency (e.g. means) or other basic estimates (e.g. regression coefficient) AND variation (e.g. standard deviation) or associated estimates of uncertainty (e.g. confidence intervals)
☐	☒	For null hypothesis testing, the test statistic (e.g. *F*, *t*, *r*) with confidence intervals, effect sizes, degrees of freedom and *P* value noted<br>*Give P values as exact values whenever suitable.*
☒	☐	For Bayesian analysis, information on the choice of priors and Markov chain Monte Carlo settings
☒	☐	For hierarchical and complex designs, identification of the appropriate level for tests and full reporting of outcomes
☐	☒	Estimates of effect sizes (e.g. Cohen's *d*, Pearson's *r*), indicating how they were calculated

Our web collection on statistics for biologists contains articles on many of the points above.

Software and code

Policy information about availability of computer code

Data collection	No custom software was involved in the collection of data.
Data analysis	Custom computational analysis was carried out by code or input in CellProfiler (v 4.2.8), Python (v 3.9), MATLAB (v R2024a), SAILOR (v 1.1.0), samtools (v 1.3.1), STAR aligner (v 2.7.6a), subread (v 1.5.3), DESeq2 (v 1.39.3), RNAlysis (v 3.9.2), Gene Set Enrichment Analysis (v 4.3.2), Metascape (v 3.5.20240101), MAJIQ and VOILA (v 2.5), and GelAnalyzer (v 19.1). When appropriate, algorithmic descriptions of analyses are given. Equivalent analyses are described in published literature. Critical code used for data analysis can be accessed at the following reference Zenodo repository: aargon11, & Brian Yee. (2025). YeoLab/Yeo_RNA_base_editing: v1.0 (v1.0). Zenodo. https://doi.org/10.5281/zenodo.16755321

For manuscripts utilizing custom algorithms or software that are central to the research but not yet described in published literature, software must be made available to editors and reviewers. We strongly encourage code deposition in a community repository (e.g. GitHub). See the Nature Portfolio guidelines for submitting code & software for further information.

Data

Policy information about availability of data

All manuscripts must include a data availability statement. This statement should provide the following information, where applicable:

- Accession codes, unique identifiers, or web links for publicly available datasets
- A description of any restrictions on data availability
- For clinical datasets or third party data, please ensure that the statement adheres to our policy

RNA-seq data from this study are available at the National Center for Biotechnology Information's Gene Expression Omnibus under GEO accession GSE295421. Datasets from GENCODE Human Release 44 (GRCh38.p14) were used in this study. Uncropped scans of gels for Fig. 4 annotated with conditions and biological replicates are provided in Supplementary Figs. 4 and 5 and separately as source data.

Human research participants

Policy information about studies involving human research participants and Sex and Gender in Research.

Reporting on sex and gender	Our study does not include human research participants.
Population characteristics	Our study does not include human research participants.
Recruitment	Our study does not include human research participants.
Ethics oversight	Our study does not include human research participants.

Note that full information on the approval of the study protocol must also be provided in the manuscript.

Field-specific reporting

Please select the one below that is the best fit for your research. If you are not sure, read the appropriate sections before making your selection.

☒ Life sciences ☐ Behavioural & social sciences ☐ Ecological, evolutionary & environmental sciences

For a reference copy of the document with all sections, see nature.com/documents/nr-reporting-summary-flat.pdf

Life sciences study design

All studies must disclose on these points even when the disclosure is negative.

Sample size	Sample sizes were determined according to the standards of the field and indicated by discrete and clearly plotted points in figures (see also Replication section). Typically n=3 biological replicates were used as is accepted in molecular and cellular biology studies. Exceptions include RNA-Seq, for which n=2 biological replicates is typical in differential gene expression analysis, and luciferase assays and lentiviral transduction, for which n=4 biological replicates is typical due to higher experimental variability.
Data exclusions	We did not exclude any data under consideration, except in instances when appropriate filters, as described in the text, were applied.
Replication	Reported experimental results were replicated independently in duplicate, triplicate, or quadruplicate, consistently among replicates. For RNA synthetic standard BID-Seq and targeted amplicon CMC sequencing, technical replicates were not essential given high experimental correlation.
Randomization	We did not use randomization of samples in this study. Randomization is generally not employed in this field. All experiments were well controlled with appropriate negative controls, as described in the text. Covariate control is not relevant to our study, because appropriate experimental negative controls were used to detect potential confounding and bias.
Blinding	Investigators were not blinded to the identities of samples in this study. Blindness is generally not employed in this field. All experiments were well controlled with appropriate negative controls, as described in the text. Blinding is not relevant to our study, because none of our collected data is clinical in nature and none of our authors had preconceived notions about experimental outcomes.

Reporting for specific materials, systems and methods

We require information from authors about some types of materials, experimental systems and methods used in many studies. Here, indicate whether each material, system or method listed is relevant to your study. If you are not sure if a list item applies to your research, read the appropriate section before selecting a response.

Materials & experimental systems

n/a	Involved in the study	
☒	☐	Antibodies
☐	☒	Eukaryotic cell lines
☒	☐	Palaeontology and archaeology
☒	☐	Animals and other organisms
☒	☐	Clinical data
☒	☐	Dual use research of concern

Methods

n/a	Involved in the study	
☒	☐	ChIP-seq
☒	☐	Flow cytometry
☒	☐	MRI-based neuroimaging

Eukaryotic cell lines

Policy information about cell lines and Sex and Gender in Research

Cell line source(s)	The Lenti-X 293T (HEK 293T) cell line (632180, Takara Bio), U-2 OS cell line (HTB-96, ATCC) , and 16HBE14o- CFTR-W1282X cell line (Cystic Fibrosis Foundation) were used in this study.
Authentication	Cell lines have been authenticated by the vendors and have been re-authenticated by STR assay prior to cryobanking in the lab. 16HBE14o- CFTR-W1282X were genotyped by Sanger sequencing of PCR-amplified mutation locus.
Mycoplasma contamination	Mycoplasma testing is routinely performed every 3 passages. All cells used in this study tested negative for mycoplasma contamination.
Commonly misidentified lines (See ICLAC register)	No commonly misidentified cell lines were used in the study.
