# Supplementary figures and images for: Enhancing RNA base editing on mammalian transcripts with small nuclear RNAs
Source: Nat Chem Biol. 2025 Sep 18;22(6):995–1003. doi: 10.1038/s41589-025-02026-8 (PMC13003931; doi:10.1038/s41589-025-02026-8)

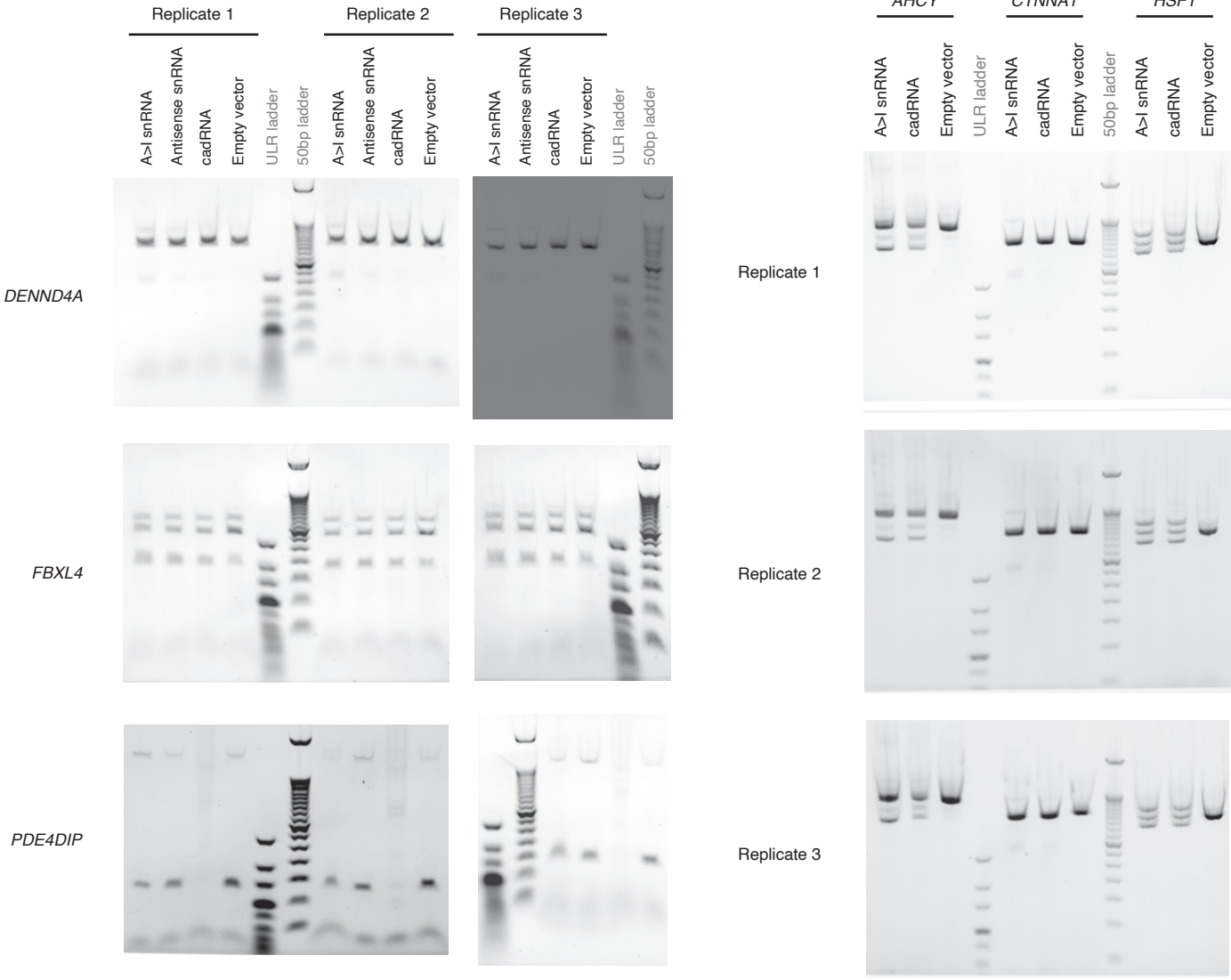

| 50bp ladder bands |     |
|-------------------|-----|
| 2500              |     |
| 800               |     |
| 750               |     |
| 700               |     |
| 650               |     |
| 600               |     |
| 550               |     |
| 500               |     |
| 450               |     |
| 400               |     |
| 350               |     |
| 300               |     |
| 250               |     |
| 200               |     |
| 150               |     |
| 100               |     |
| 50                |     |
| ULR ladder bands  |     |
|                   | 300 |
|                   | 200 |
|                   | 150 |
|                   | 100 |
|                   | 75  |
|                   | 50  |
|                   | 35  |
|                   | 20  |
|                   | 10  |

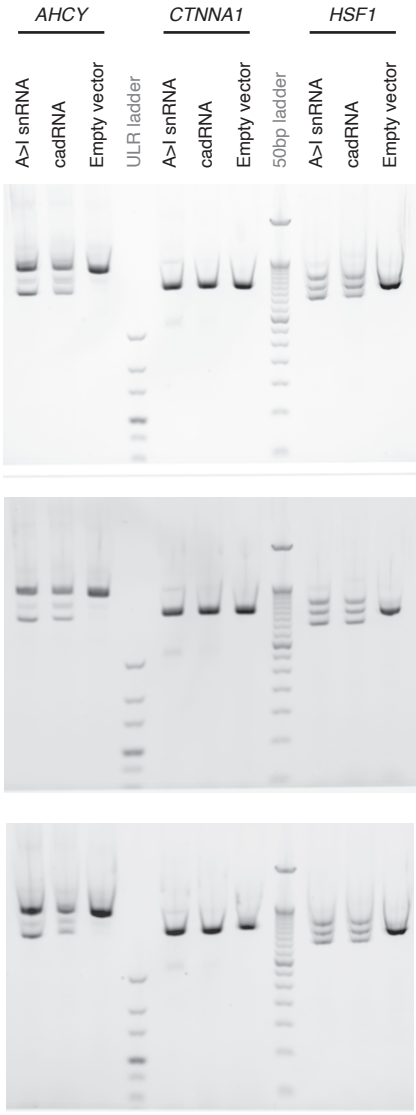

Supplement: Supplementary file 4 — Uncropped scans of gels for Fig. 4. [file 41589_2025_2026_MOESM4_ESM.pdf]
